# Structural optimization of different truss designs using two archive multi objective crystal structure optimization algorithm

**DOI:** 10.1038/s41598-025-97133-w

**Published:** 2025-04-25

**Authors:** Pranav Mehta, Ghanshyam G. Tejani, Seyed Jalaleddin Mousavirad

**Affiliations:** 1 Department of Mechanical Engineering, Dharmsinh Desai University, Nadiad, Gujarat 387001 India; 2 Department of Research Analytics, Saveetha Dental College and Hospitals, Saveetha Institute of Medical and Technical Sciences, Saveetha University, Chennai, 600077 India; 3 Department of Industrial Engineering and Management, Yuan Ze University, Taoyuan, 320315 Taiwan; 4 Department of Computer and Electrical Engineering, Mid Sweden University, Sundsvall, 851 70 Sweden

**Keywords:** Multi-objective optimizers, 2-archives, Truss optimization, Pareto-fronts, Applied mathematics, Mechanical engineering

## Abstract

Optimizing a multi-objective structure is a challenging design problem that requires handling several competing goals and constraints. Despite their success in resolving such issues, metaheuristics can be difficult to apply due to their stochastic nature and restrictions. This work proposes the multi-objective crystal structure optimizer (MOCRY), a potent and effective optimizer, to address this problem. The MOCRY algorithm, also known as MOCRY2arc, is built on a two-archive idea centered on diversity and convergence, respectively. The efficacy of MOCRY2arc in solving five typical planar and spatial real-world structure optimization issues was assessed. Because of these problems, safety and size limits were put on discrete cross-sectional regions and component stress. At the same time, different goals were being pursued, such as making nodal points bend more and reducing the mass of trusses. Four recognized standard evaluators—Hypervolume (HV), Generational-Inverted Generational Distance (GD, IGD), Spacing to Extent Metrics (STE), convergence, and diversity plots—were utilized to compare the results with those of nine sophisticated optimization techniques, including MOCRY and NSGA-II. Moreover, the Friedman rank test and comparison analysis showed that MOCRY2arc performed better at resolving big structure optimization issues in a shorter amount of computing time. In addition to identifying and realizing effective Pareto-optimal sets, the recommended method produced strong variety and convergence in the objective and choice spaces. As a result, MOCRY2arc may be a useful tool for handling challenging multi-objective structure optimization issues.

## Introduction

In recent times, the world has been transforming from conventional methods for product design or development to novel artificial intelligence and machine learning techniques. These techniques have the potential to enhance product standards and enable access to the development or design domain from anywhere in the world. Optimization techniques play a crucial role in industries and research organizations, facilitating the optimization of products in terms of cost, dimensions, parameters, and time consumption. Consequently, for many years, classical optimization algorithms have served as viable options for optimizing various engineering and multidisciplinary tasks. Additionally, a wide range of design optimization problems, from the product development range to the finalization stage, utilize classical optimization techniques^1–3^. Efficient and effective design and product development necessitate the development of further potential techniques capable of handling multi-modal problems, critical constraints, and the non-linear nature of functions, all while achieving the best globally optimized solutions. Consequently, a trend of nature-inspired algorithms has emerged, offering innovative alternatives to traditional optimization algorithms. These algorithms that are based on nature are usually called metaheuristics (MHs) algorithms. They are known to be more efficient and proven convergence solutions that keep a good balance between the exploration and exploitation phases and can handle critical constraint functions^3^. We further incorporate these MHs with various techniques to enhance the solution quality, including oppositional-based learning techniques^4^, chaotic maps^5^, the Levy flight mechanism^6^, elite oppositional-based learning techniques^7^, and hybridized two MHs optimizers^8^. In addition, researchers developed and applied multi-objective versions of various MHs to simultaneously optimize multiple fitness functions across a wide range of applications. The initial focus of the multi-objective optimizers was on the Pareto fronts, which are non-dominated solution sets that provide a converged global optimized solution for fitness functions. The accuracy and effectiveness of the multi-objective optimizer can be realized by identifying the nature of Pareto fronts, diversity, and several other metrics^9–12^. Several applications were observed that utilized novel multi-objective optimization algorithms, such as not limited to but including truss structure optimization^13^, solution of EDM problems^14^, real-world engineering problems^15^, multi-factorial optimization problems^16^, and fuzzy circuits. Moreover, truss structures are imperative elements of the civil engineering discipline that provide potential support for various applications, such as stadiums, cranes, electrical transmission towers, wind turbine towers, and mechanical systems. In addition, standard agencies like the American Society of Civil Engineers (ASCE) have imposed constraints and standards on structural optimization, primarily to reduce overall weight or cost. Therefore, the adoption of multi-objective optimizers results in the realization of more efficient design and structural parameters. Researchers in the truss optimization domain developed potential MOMHs using various techniques and verification standards to optimize two fitness functions: the minimum weight of the overall structures and the minimization of the maximum nodal deflection of the elements.

In one of the studies, a hybrid symmetric laminated composite structures have been optimized by considering the uncertain buckling load effect. The study was conducted by applying a unique approach of Quantum inspired evolutionary algorithm. Moreover, the study further investigates the different configurations of the structure under different loading conditions, aspect ratio and material properties^17^. In another study, a NSGA-II was applied to manage the initial and seismic damage cost of the steel structure. Moreover, the computational time is reduced by utilizing the stated approach with generalized regression neural network for controlling several parameters^18^. The reinforced concrete structures are optimized in terms of cost and minimum emission of carbon dioxide gases while in operation. For attaining these objectives enhanced vibration particles system, modified-colliding bodies optimizers and particle swarm algorithm have been utilized^19^. Apart from this multi-objective optimization algorithms have been employed to optimize the steel structures and reinforced concrete wall structures^20,21^. Multi objective optimization of different truss structures have been attained using Mult objective version of the vibrating particle system and results are compared with other well-known multi objective optimizers. The Pareto-fronts identified by the studies MO optimizer found potential^22^. Furthermore, several state-of-the-art studies demonstrates MO and modified versions of the optimization algorithms for truss optimization. For instance, multi-objective charged system search^23^, hybrid multi-objective particle swarm optimizer^24^, multi-objective cuckoo search algorithm^25^, evolutionary graph-based multi-objective algorithm^26^, multi-swarm multi-objective optimizer^27^and multi-objective colliding bodies optimizer^28^.

In the recent times, authors potentially identified that nature inspired optimizers may sometimes leads to poor quality results and unable to identified global optimize solutions. One of the major reasons behind that is the metaphor-based optimizers. Accordingly, the justifications demand regarding the scientific concerns related to the metaphor-based algorithms^29^. Moreover, another study identified six metaphor-based metaheuristics that developed based on the particle swarm or evolutionary-based techniques. Moreover, the authors present different components of the developed optimizers that gives an effective way to understood the no-free-lunch theorems for optimization in a better way^30^. Several studies also claimed that whale optimization algorithm and arithmetic optimizer contain center-bias operator that realized ineffective results. However, the exploration phase of the metaphor-based algorithm may be unsuitable for attaining the global optimum solutions^31,32^.

Researchers have made several additions to the optimizer in the MOMHs research domain to enhance its performance and effectiveness. For instance, we have implemented two archive techniques to enhance the algorithm’s population diversity while simultaneously converging the solution to the Pareto fronts. Additionally, we have implemented the multi-strategy and multi-model approach, many objectives, and the external archive-based approach as potential methods to further enhance the performance of the MOMHs algorithms. So, this study presents a brand-new two-archive multi-objective crystal structure algorithm (MOCRY2arc) for improving the structure of eight constrained truss structures. Furthermore, the lattice structure and crystal growth at the atomic and molecular levels served as inspiration for the development of the crystal structure algorithm. The state-of-the-art algorithm aimed to pursue the following objectives:

- The aim of the study is to identify the effectiveness of the MOCRY for the global optimization of various truss structures by incorporating two archives strategy. Accordingly, the results can be justified by taking acceptable balance between the exploration and exploitation phase of the algorithm. Accordingly, two-archive based MOCRY algorithm compared with relevant competitors for justifications of results trends and statistics.
- The goal of this study was to find the best eight truss structures, mostly 10-, 25-, 37-, 60-, 72-, 120-, 200-, and 942-bar ones, by lowering their maximum nodal deflection and lowering their minimum structural mass.
- The execution of MOCRY2arc was compared with nine benchmark algorithms. The other competitive optimizers selected for the performance assessments are MOALO^12^, MOCRY^33^, MOBA^34^, NSGA-II^35^, DEMO^36^, MSSA^37^, MODA^38^, and MOWCA^39^.
- Accordingly, the statistical test of the proposed optimizer are realized, such as Hypervolume tests (HV), spacing-to-extent tests (STE), generational and inverted-generational distance metrics (GD and IGD), and Friedman’s rank test on an average and global basis.
- The study identifies a potential option within the research domain of multi-objective optimization algorithms for addressing critical constrained truss structure issues. It provides a strong competitive comparison among benchmark optimizers, which could further enhance the effectiveness and versatility of each algorithm.

## Multi-Objective crystal structure (MOCRY) algorithm Understanding

The CRY optimizer draws its motivation from the naturally occurring and unique crystal structure of quartz. The motivation for the development of the algorithm is based on the structural development and growth of the crystal at the atomic and molecular level. The main objective of the CRY algorithm is the existence of the lattice point in nature, which follows the development of the crystal structure. Apart from this, Galena is also a naturally identified crystal structure, which exists in multiple-layered configurations as BCC (body-centered cubic), HCC (hexagonal cubic center), and FCC (face-centered cubic). The crystallographic configuration in terms of population development, basis development, and different structure growth that enables the optimizer to attain the global optimum solution is mathematically modeled as follows. Equation (1)^33^ develops the initial location of the crystals in the lattice, further modeling each crystal as an individual candidate solution.

1 $$\:{y}_{i}^{j}\left(0\right)=\:{y}_{i,\:min}^{j}+\xi\:\:({y}_{i,\:max}^{j}-{y}_{i,\:min}^{j})$$

where, $\:\xi\:$ is random number ranging from 0 to 1, with provisional location of the crystal is $\:{y}_{i}^{j}\left(0\right)$ relative to $\:j$-th variable for $\:i$-the iteration. Accordingly, $\:{y}_{i,\:min}^{j}$ and $\:{y}_{i,\:max}^{j}$ are extreme limits of the design variables.

Here, the term “basis” refers to the process of developing an individual crystal structure. This process involves allocating four different configurations to identify the best candidate solution, ultimately leading to the global optimum solution. For instance, crystal structures with cubicles, effective crystals with cubicles, mean crystal configurations with cubicles detailing, and superior and mean crystal systems with cubicles detailing. This is being modeled as per the Eqs. (2–5).

SCC—simple cubic structure—can be modeled as follows^33^:

2 $$\:{Cr}_{new}={Cr}_{old}+r{Cr}_{main}$$

FCC and HCP configurations can be modeled as follows^33^:

3 $$\:{Cr}_{new}={Cr}_{old}+{r}_{1}{Cr}_{main}+{r}_{2}{Cr}_{b}$$

4 $$\:{Cr}_{new}={Cr}_{old}+{r}_{1}{Cr}_{main}+{r}_{2}{F}_{c}$$

5 $$\:{Cr}_{new}={Cr}_{old}+{r}_{1}{Cr}_{main}+{r}_{2}{Cr}_{b}+{r}_{3}{F}_{c}$$

where, $\:{Cr}_{new}$,$\:\:{Cr}_{b}$ and $\:{Cr}_{old}$ refers to latest location of the crystal, best crystal and recent location of the crystal respectively with randomly identified crystal is denoted as $\:{F}_{c}$ subsequently, a significant balance has been realized between exploration and exploitation phase once the algorithm is executed with the Eqs. (2–4).

The considered algorithm can be applied to optimize single fitness function problems. However, for attaining the optimal solution of a problem that consists of multiple objective functions, a multi-objective version is required. Hence therefore, the current study broadens the development of a multi-objective version of the CRY algorithm, concentrating on implementing a non-dominated solution (NDS) approach in conjunction with dominance theory. In order to identify the non-dominated solution sets, two solutions are compared in order to determine which is the best and which is the worst. To find non-dominated solution sets, the function vectors of two design solutions are compared. When two functional vector sets are chosen from among the available elements, at least one element in one set must match another set in order to meet the mathematical criterion. A collection of non-dominated solutions is then sought for based on the solutions that are not dominated by other solutions. MOCRY updated external archives with non-dominated solutions using the ε-dominance technique. Additionally, MOCRY divides the solutions into squares and boxes based on the number of objective functions. To ensure that only non-dominated solutions are kept in each categorized box, dominated solutions are eliminated. The grid-based method saves the best answer in a fixed-size archive every time the algorithm updates. Furthermore, at each iteration step, the -dominance technique updates the archive, preserving only non-dominance solutions within it. In light of this, MOCRY is able to efficiently search the domain in order to find the best solutions worldwide.

## A two-archive multi-objective crystal structure algorithm (MOCRY2 Arc)

The MOCRY optimizer employs a two-archive strategy to enhance convergence towards Pareto fronts while maintaining population diversity. In each update phase, the optimizer classifies a set of non-dominated solutions (NDSs) into two archives, eliminating dominated solutions and preserving only the non-dominated ones. The newly generated solution is then assessed against the stored solutions based on three verticals. In the first scenario, it is assumed that the existing solution sets either do not impact the superior outcome or influence other members within the archive. In the second scenario, both existing and newly introduced solutions remain independent without interaction. The third case results in the rejection of a new solution as it violates the archival property boundary. When a new solution outperforms a previous one, it is incorporated into the archive, with younger members demonstrating superior performance over older ones. The archiving process remains unaffected before and after collection in the second scenario. Once fresh solutions are stored, MOCRY processes each member uniformly, and if the archive reaches its capacity, any member may be removed to maintain balance. Two-archive strategies are incorporated into multi-objective optimization algorithms to enhance both convergence towards the Pareto front and population diversity. The primary reasons for using two archives in these algorithms are:

### Exploration and exploitation

While one archive preserves diverse solutions to cover a wider search space (exploration), the other archive concentrates on preserving high-quality, well-converged solutions close to the Pareto front (exploitation). This equilibrium guarantees a well-distributed set of optimal solutions and avoids premature convergence.

### Effective selection and updating

The algorithm able to filter and update non-dominated solutions in a methodical manner by classifying solutions into two archives. This guarantees that only the most pertinent solutions are kept for the following iteration while preventing the loss of potentially helpful ones.

### Managing archival constraints

Using two archives offers an organized method of dealing with small archive sizes. While the other archive concentrates on diversity to avoid overcrowding in particular areas of the solution space, the first archive may rank elite solutions according on convergence quality.

### Better performance in dynamic environments

Keeping two archives enables the algorithm to adjust by monitoring previous solutions while investigating novel possibilities, improving flexibility and robustness in dynamic multi-objective situations where objectives vary over time.

The solution set undergoes further segmentation into two archives. If the new solution outperforms each member, we remove it from the first set of archives. In Archive- 2, multiple scenarios persist, including the removal of a member from the archive due to capacity overflow and the dominance of a new solution over the existing one. The selection strategy for identifying the leader is based on the probability of the main leader being the MOCRY, or alternatively, it can be selected from the three available strategies in Archives- 2. The algorithm handles the exploitation phase by generating Archive- 1. The algorithm then generates the leader from the available set and exponentially increases its value, as demonstrated in Eq. (6)^40^.

6 $$\:{L}_{P}\left(t\right)={c}_{1}\:\times\:{e}^{{c}_{2}t}$$

$$\:{c}_{1}=\:\frac{{L}_{ps}}{{e}^{{c}_{1}}}\:{c}_{2}=\frac{\text{ln}\left({L}_{ps}\right)\text{l}\text{n}\left({L}_{pf}\right)}{{t}_{max}-1}$$

where, initial and final point values of probability is indicated with $\:{L}_{ps}$ and $\:{L}_{pf}$ respectively.

**Fig. 1:**
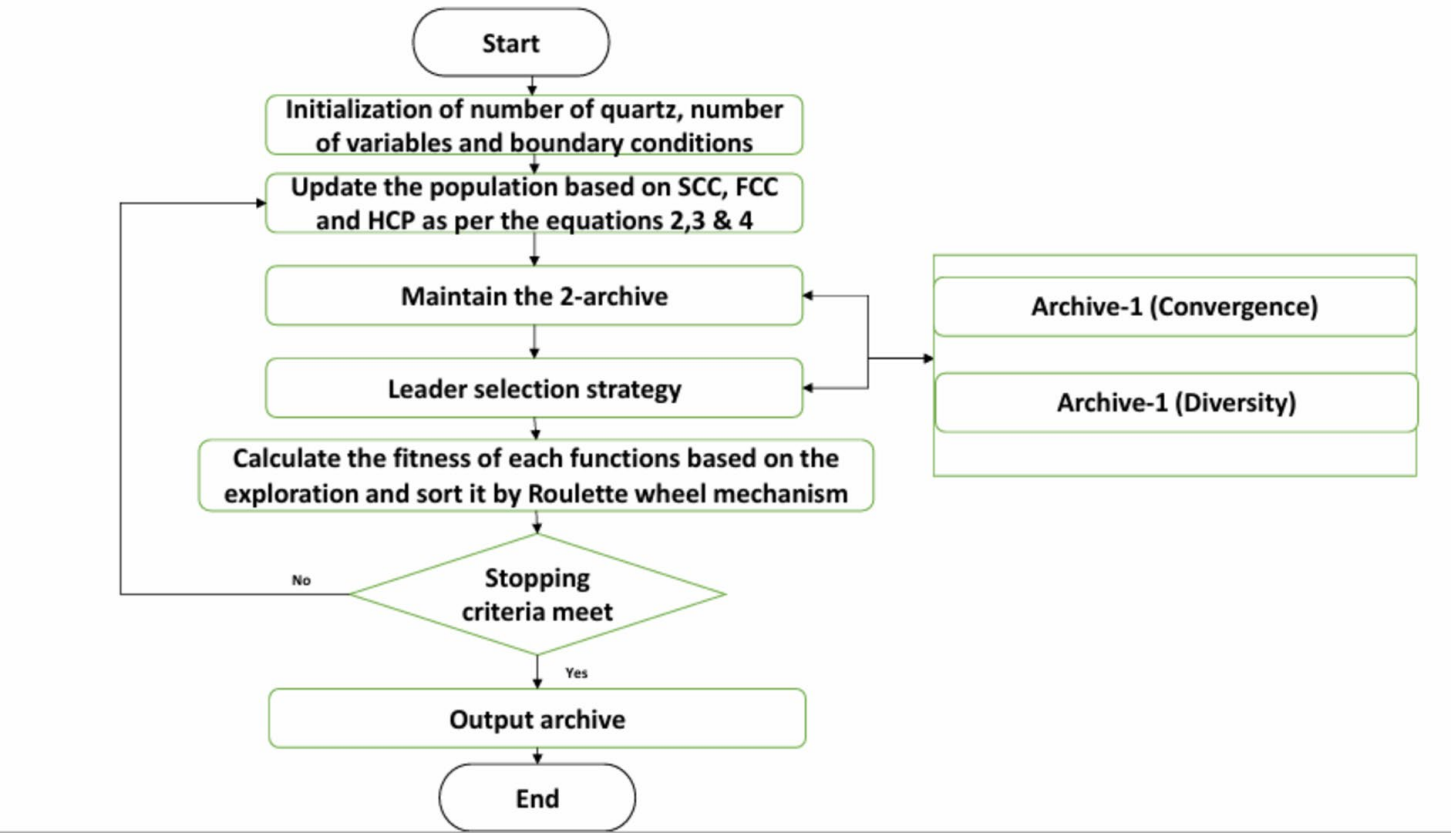
Flow chart of MOCRY2 Arc.

In the archives- 2 generation, there are two surplus objective functions generated based on the NDSs stored in the archives. For the solution $\:{P}_{i}$ in the solution set, a first objective aligns with the diverse solution that implicating population diversity indicator in the algorithm. Furthermore, the second objective function employs the weighted sum of multi-objective optimizers, which generates weighted factors at random solutions to preserve diversity. Consequently, the first objective and second secondary objective functions, respectively, balance exploration and exploitation. Equations (7–8)^40^ model both exploitation and exploration.

7 $$\:{f}_{1}^{{\prime\:}}\left({P}_{i}\right)=\:\frac{1}{\sum\:_{j=1}^{{N}_{2}}\|{f}_{i}-{f}_{j}\|}$$

8 $$\:{f}_{2}^{{\prime\:}}\left({P}_{i}\right)={W}_{g}^{T}\:{f}_{i}$$

where, total numbers of design solutions and objective function is indicated with $\:{N}_{2}$ and $\:{f}_{i}$ respectively. The weight factor vector is denoted with $\:{W}_{g}^{T}$. For selection of leaders MOCRY2arc utilized archive- 2. Leader- 1 is designated for the first iteration following the creation of the original population and both archives. The universes’ orientation

is modified in accordance with the first MOCRY. Additionally, the likelihood $\:{L}_{p}$ is increasing exponentially, and both archives are updated with fresh universe positions. The procedure is repeated until the termination requirements are met. The flowchart of the proposed optimizer can be depicted in Fig. 1.

## Objective functions and constraints

The present study handles two objective functions: structural mass and nodal deflections with multiple critical constraints in the form of load, stress, and deflections. Moreover, we aim to minimize the structural mass of the study’s truss bars and the maximum nodal deflections of their elements while simultaneously managing the constraints. Table 1 provides the design configurations. Equation (9)^40^ accordingly gives the problem’s definition in the computational domain.

**Table 1:** Design considerations of the truss problems.

	The 10-bar truss	The 25-bar truss	The 37-bar truss	The 60-bar truss	The 72-bar truss	The 120-bar truss	The 200-bar truss	The 942-bar truss
Design variables	$$\begin{aligned} &{A}_{i,}i\\&=\text{1,2},\dots ,10 \end{aligned}$$	$$\begin{aligned} &{A}_{i,}i\\&=\text{1,2},\dots ,8 \end{aligned}$$	$$\begin{aligned} &{A}_{i,}i\\&=\text{1,2},\dots ,15 \end{aligned}$$	$$\begin{aligned} &{A}_{i,}i\\&=\text{1,2},\dots ,25\end{aligned}$$	$$\begin{aligned} &{A}_{i,}i\\&=\text{1,2},\dots ,16\end{aligned}$$	$$\begin{aligned} &{A}_{i,}i\\&=\text{1,2},\dots ,7\end{aligned}$$	$$\begin{aligned} &{A}_{i,}i\\&=\text{1,2},\dots ,29\end{aligned}$$	$$\begin{aligned} &{A}_{i,}i\\&=\text{1,2},\dots ,59\end{aligned}$$
Stress Constraints	σ^max^ = 400 MPa	σ^max^ = 400 MPa	σ^max^ = 400 MPa	σ^max^ = 400 MPa	σ^max^ = 400 MPa	σ^max^ = 400 MPa	σ^max^ = 400 MPa	σ^max^ = 400 MPa
Density	ρ = 7850 kg/m^3^	ρ = 7850 kg/m^3^	ρ = 7850 kg/m^3^	ρ = 7850 kg/m^3^	ρ = 7850 kg/m^3^	ρ = 7850 kg/m^3^	ρ = 7850 kg/m^3^	ρ = 7850 kg/m^3^
Young’s modulus	E = 200 GPA	E = 200 GPA	E = 200 GPA	E = 200 GPA	E = 200 GPA	E = 200 GPA	E = 200 GPA	E = 200 GPA
Size variables	$$\begin{aligned} &{A}_{i,}\in\:S, \\& i=\text{1,2},\dots ,10\end{aligned}$$ *S*=[1, 1.5, 2, …,21] * 1e- 3 m^2^	$$\begin{aligned} &{A}_{i,}\in S, \\& i= \text{1,2},\dots ,8\end{aligned}$$ *S*=[1, 1.5, 2,…,21] * 1e- 3 m^2^	${A}_{i,} \in S, i= \text{1,2}, \dots ,8$ *S*=[1, 1.5, 2,…,21] * 1e- 3 m^2^	${A}_{i,}\in S, i=1,2, \dots ,8$ *S*=[1, 1.5, 2, …,21] * 1e- 3 m^2^	${A}_{i,}\in S, i= \text{1,2}, \dots ,8$ *S*=[1, 1.5, 2,…,21] * 1e- 3 m^2^	${A}_{i,}\in S, i= \text{1,2}, \dots ,8$ *S*=[1, 1.5, 2, …,21] * 1e- 3 m^2^	${A}_{i,}\in S, i= \text{1,2}, \dots ,8$ *S*=[1, 1.5, 2,…,21] * 1e- 3 m^2^	${A}_{i,}\in S, i = \text{1,2}, \dots ,59$ *S*=[1, 1.5, 2, …,21] * 1e- 1 m^2^
Loading conditions	$$\begin{aligned} &{P}_{y2}={P}_{y4}=\\& -1000\:\text{KN}\end{aligned}$$	${P}_{x1}=100\:\text{KN},$ $$\begin{aligned} &{P}_{y1}={P}_{z1}={P}_{y2}= {P}_{z2}\\&=-1000\: \text{KN},\end{aligned}$$ $$\begin{aligned} &{P}_{x3}=50\:\text{KN}, \\&{P}_{x6} = 60\: \text{K}\text{N}\end{aligned}$$	${P}_{y2},{P}_{y3},{P}_{y4},\dots ,$ $$\begin{aligned} &{P}_{\text{y}10} =\\& -100\: \text{KN}\end{aligned}$$	$$\begin{aligned} &{\text{Case 1:}}\\&{P}_{x1}=-1000\:\text{KN},\\&{P}_{x7}=900\: \text{KN}\end{aligned}$$ $$\begin{aligned} &{\text{Case 2:}}\\&{P}_{x15}={P}_{x18}=-800 \: \text{KN},\end{aligned}$$ ${P}_{y15}={P}_{y18}=300\:\text{KN}$ $$\begin{aligned} &{\text{Case 3:}}\\&{P}_{x22}=-2000\: \text{KN and}\end{aligned}$$ ${P}_{y22}=1000\:\text{KN}$	$$\begin{aligned} &{\text{Case 1:}}\\&{F}_{17x}={F}_{17y}\\&=2000\:\text{K}\text{N} ,\end{aligned}$$ ${F}_{17z}=-2000\:\text{K}\text{N}$ $$\begin{aligned} &{\text{Case 2:}}\\&{F}_{17z}={F}_{18z}={F}_{19z}\\&={F}_{20z}=-2000\:\text{KN}\end{aligned}$$	${P}_{z28},{P}_{z29},{P}_{z30},\dots \: ,$ ${P}_{z36} = -500 \: \text{KN} ,$ ${P}_{z37},{P}_{z38},{P}_{z39},\dots \: ,$ ${P}_{z48} = -1500 \: \text{KN}$, ${P}_{z49} = -3000 \: \text{KN}$	${P}_{x1},{P}_{x6},{P}_{x15},$ ${P}_{x20},{P}_{x29},{P}_{x34},$ ${P}_{x43},{P}_{x48},{P}_{x57},$ ${P}_{x71}=10 \: \text{KN}$ ${P}_{y1},{P}_{y2},\dots\:,{P}_{y6},$ ${P}_{y8},{P}_{y10},{P}_{y12},$ ${P}_{y14},{P}_{y15}, \dots ,{P}_{y20}$ ${P}_{y22},{P}_{y24},{P}_{y26},$ $$\begin{aligned} &{P}_{y28},{P}_{y29},\dots ,\\& {P}_{y34},{P}_{y36},{P}_{y40},\end{aligned}$$ ${P}_{y36},{P}_{y38},{P}_{y40},$ ${P}_{y42},{P}_{y43}, \dots\:, {P}_{y48}$ ${P}_{y50},{P}_{y52},{P}_{y54},$ ${P}_{y56},{P}_{y57}, \dots , {P}_{y62},$ ${P}_{y64},{P}_{y66},{P}_{y68},$ ${P}_{y70},\:{P}_{y71}, \dots , {P}_{y75},$ $=-100\:\text{KN}$	At each node: vertical loading: Section 1; ${P}_{z}=-6\:\text{K}\text{N}$ Section 2; ${P}_{z}=-12\:\text{K}\text{N}$ Section 3; ${P}_{z}=-18\:\text{K}\text{N}$ Lateral loading: Right-hand side; ${P}_{x}=3\:\text{K}\text{N}$ Left-hand side; ${P}_{x}=2\:\text{K}\text{N}$ Lateral Loading: ${P}_{y}=2\:\text{K}\text{N}$

Find $$\:X=\:\left\{{X}_{1},{X}_{2},{X}_{3}\dots\:\dots\:\dots\:.,{X}_{m}\right\}$$

Structural mass- first objective with target to minimize

9 $$\:{f}_{1}\left(X\right)=\:\sum\:_{i=1}^{m}{X}_{i}{\rho\:}_{i}{L}_{i}$$

Nodal deflection- second objective with target to minimize

$$\:{f}_{1}\left(X\right)=\text{m}\text{a}\text{x}\left({\delta\:}_{j}\right)$$

Constraints: stress (tensile and compressive) and cross-sectional area.

Constraint 1: $$\:\left|{\sigma\:}_{i}\right|-{\sigma\:}_{i}^{max}\le\:0$$

Constraint 2: $$\:{X}_{i}^{min}\le\:{X}_{i}\le\:{X}_{i}^{max}$$

where, cross-sectional area vector, mass density and length of the truss bar is denoted with $\:X,{\rho\:}_{i}$ and $\:{L}_{i}$ respectively. Moreover, the dynamic penalty function to address the constraints requirement is given by Eq. (10).

10 $$\:{f}_{penalty}\left(X\right)=\:\left\{\begin{array}{c}f\:\left(X\right)\\\:no\:constraints\:violations\end{array}\right.$$

$$\:f\:\left(X\right)={(1+{\in\:}_{1}\times\:C)}^{{\in\:}_{2}}$$

$$\:C=\:\sum\:_{i=1}^{q}{C}_{i},\:{C}_{i}=\:\left|1-\frac{{P}_{i}}{{P}_{i}^{*}}\right|$$

where, $\:{P}_{i}$ and $\:{P}_{i}^{*}$ is the values of constraint violation penalty and maximum penalty at i^th^ iteration respectively.

### Experimental assessments

The experiments are conducted to verify the effectiveness of MOCRY2arc optimizers for the eight complex truss structures (10-, 25-, 37-, 60-, 72-, 120-, 200-, and 942-bar). In addition to this statistical analysis, we evaluated convergence and diversity tests to identify potential Pareto front sets, and compared MOALO, MOCRY, MOBA, NSGA-II, DEMO, MSSA, MODA, MOWCA, MOBA, and MOCRY. This being said that four parameters were evaluated to potentially check the performance of the algorithms. First, indicator S shows how convergent and diverse the set S is within the search domain. It does this by showing how much space each NDS takes up in the search domain using hypervolume (HV) metrics. For an HV test, a larger number of results indicates better algorithm performance. The mathematical Eq. (11) provides details about the HV index.

11 $$\:HV=volume\:\left(\:\bigcup\:_{i=1}^{A}{V}_{i}\right)$$

The Generational Distance (GD) and Inverted Generational Distance (IGD) are performance metrics used to evaluate the quality of solutions obtained by multi-objective optimization algorithms. They measure how closely the obtained Pareto front approximates the true Pareto front. GD measures the average Euclidean distance between the solutions in the obtained Pareto front and the nearest points in the true Pareto front. A lower GD indicates that the obtained solutions are closer to the optimal front. Whereas, IGD computes the average distance from points in the true Pareto front to their closest counterparts in the obtained Pareto front. A lower IGD suggests that the obtained solutions cover the true Pareto front more effectively. Equations (12–13) provide the same information. Accordingly, lower values of GD and IGD metrics are preferred for better performance.

12 $$\:GD=\:\frac{\sqrt{\sum\:_{i=1}^{no}{d}_{i}^{2}}}{\left|P\right|}\:$$

13 $$\:IGD=\:\frac{\sqrt{\sum\:_{i=1}^{nt}{d{\prime\:}}_{i}^{2}}}{\left|P{\prime\:}\right|}$$

where, Euclidian distance is denoted with $\:di$, Pareto front solution counts are represented by $\:\left|P\right|$. Moreover, $\:\left|P{\prime\:}\right|$ represents the number of Pareto front solutions in the reference plane with $\:d{\prime\:}i$ denoted the distance between optimum solution from the previous front and fitness function vector of the i^th^ solution. Accordingly, IGD can provide diversity vis-a-vis progression of the search domain Pareto fronts. The fourth parameter for performance assessment is the Spacing to Extent (STE). This gives the parametric analysis regarding spacing between Pareto fronts and, effective fronts have the smaller values of STE metrics. The extent and spacing can be given by Eqs. (14–15) respectively.

14 $$\:SP=\:\frac{1}{\left|P\right|-1}\sum\:_{i=1}^{\left|P\right|}{({d}_{i}-\stackrel{-}{d})}^{2}$$

15 $$\:ET=\:\sum\:_{i=1}^{M}\left|{f}_{i}^{max}-{f}_{i}^{min}\right|$$

where, total counts of objective function are denoted with M. Whereas, maximum and least values of the objective function for the i^th^ Pareto front is denoted with $\:{f}_{i}^{max}$ and $\:{f}_{i}^{min}$ respectively. Moreover, average values of all d_i_ is denoted by $\:\stackrel{-}{d}$.

## Results and discussion

**Fig. 2:**
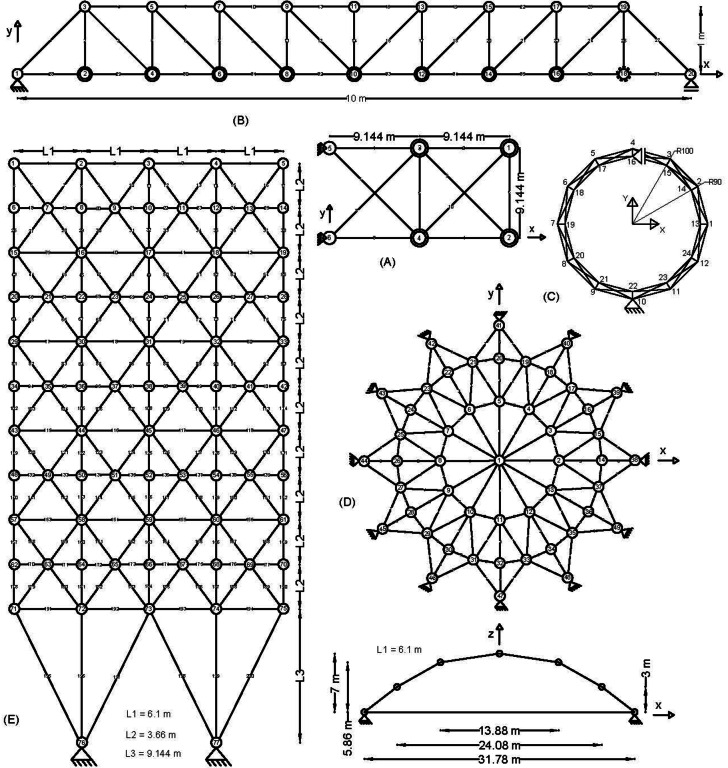
Trusses: (A) 10-bar truss, (B) 37-bar truss, (C) 60-bar truss, (D) 120-bar truss, and (E) 200-bar truss^8,13,40^.

**Fig. 3:**
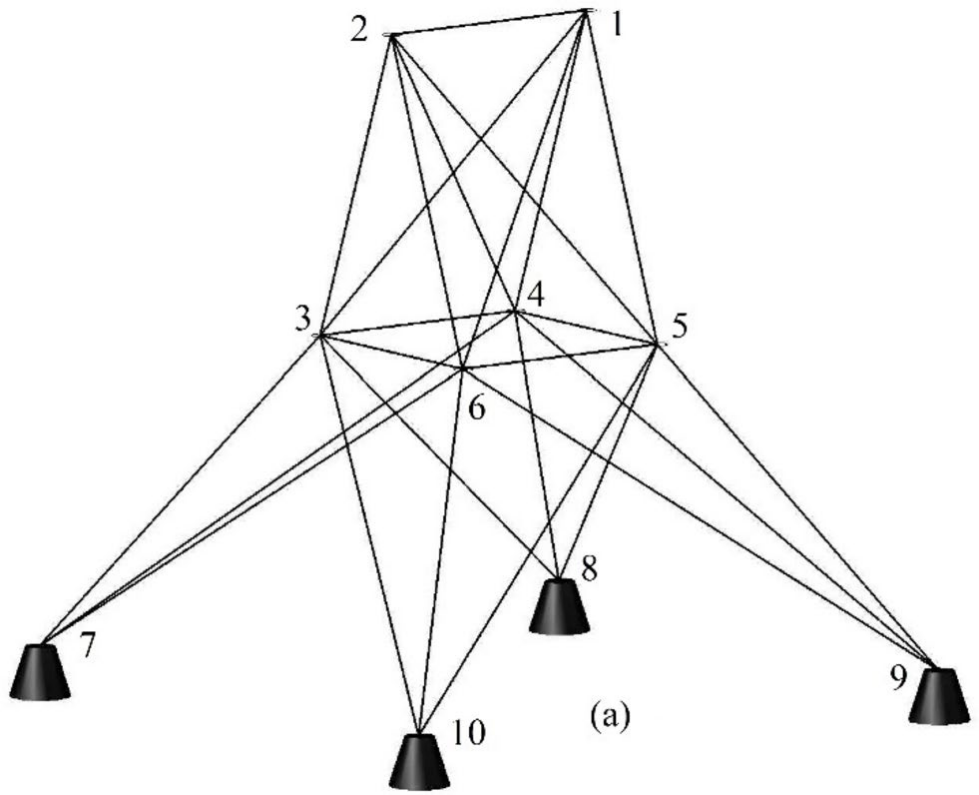
The 25-bar 3D truss.

**Fig. 4:**
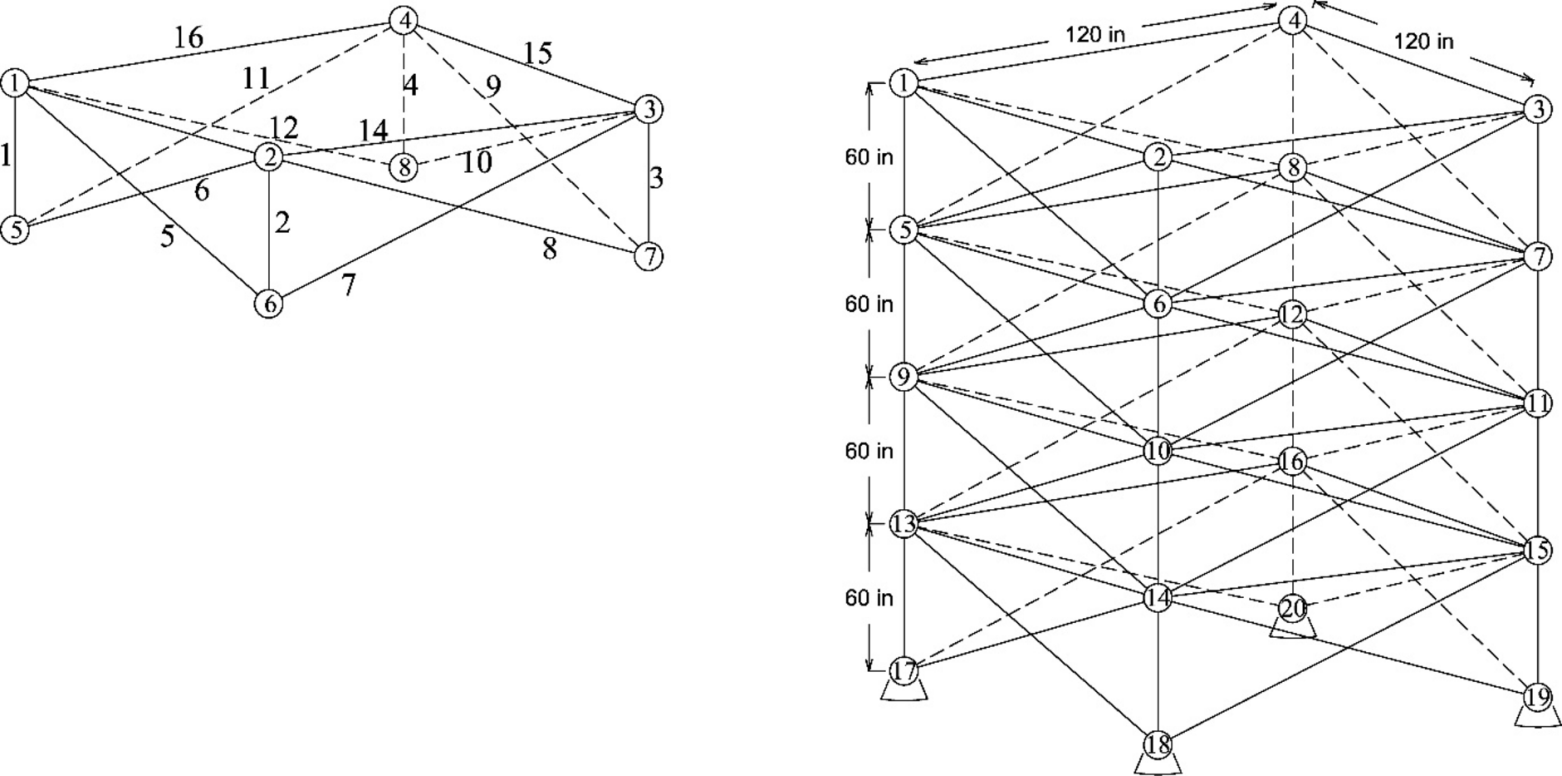
The 72-bar 3D truss^8,13,40^.

**Fig. 5:**
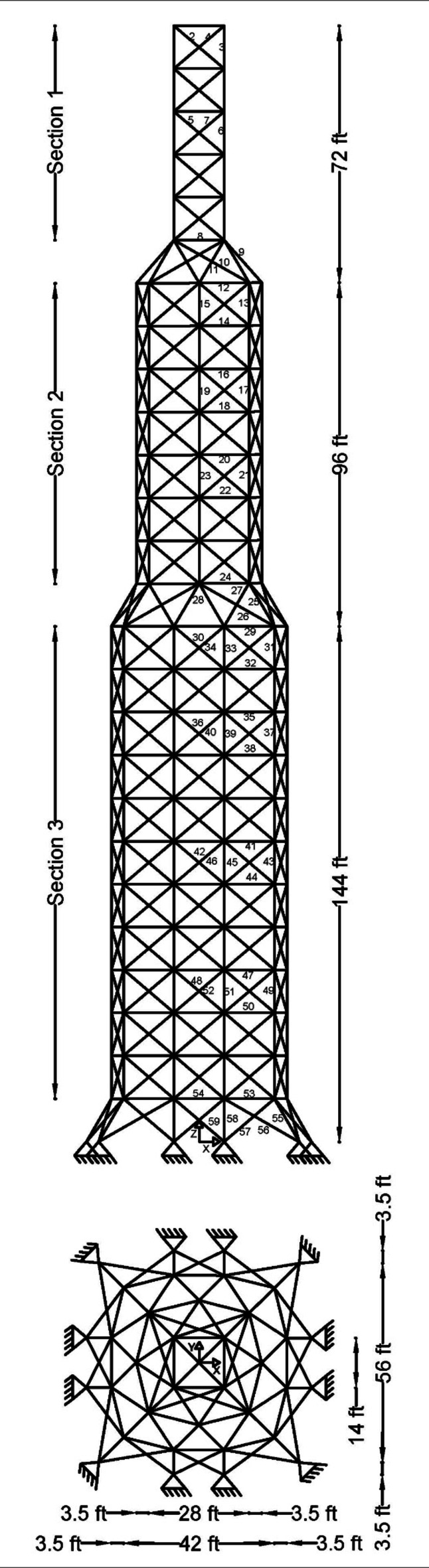
The 942-bar tower truss^8,13,40^.

Figure 2 shows the basic and most widely utilized truss designs (10-, 37-, 60-, 120-, and 200-bar) with design variables and loads acting over them. Moreover, Figs. 3 and 4 depict 3-D designs of 25-bar and 72-bar, respectively. The 942-bar truss, which consists of diamond-shaped structural elements as shown in Fig. 5, is one of the most critical and challenging truss systems to optimize. Table 1 tabulates the detailed design configurations of each truss structure, including design variables, stress constraints, loading conditions, and relevant constants. Furthermore, each tested optimizer, including MOCRY2arc, is evaluated with 50,000 functional evaluations with 100 independent tests.

Accordingly, Tables 2, 3, 4, 5 and 6 present the statistical results of performance evaluations of compared algorithms under the specified matrices.

**Table 2:** The hypervolume (HV) of the considered truss structures.

HV		MOALO	MODA	MOMVO	MOWCA	MSSA	NSGA-II	DEMO	MOBA	MOCRY2 A	MOCRY
10-bar	average	1.86E + 09	2.22E + 09	2.35E + 09	1.04E + 09	2.3E + 09	2.16E + 09	1.87E + 09	2.17E + 09	2.41E + 09	2.41E + 09
	max	2.22E + 09	2.34E + 09	2.36E + 09	1.67E + 09	2.35E + 09	2.25E + 09	2.05E + 09	2.31E + 09	**2.41E + 09**	2.41E + 09
	min	1.09E + 09	2.12E + 09	2.29E + 09	0	2.22E + 09	1.86E + 09	1.76E + 09	1.98E + 09	2.39E + 09	2.39E + 09
	std	2.61E + 08	53,390,824	13,641,612	5.32E + 08	38,548,017	81,087,395	60,920,753	79,239,356	4,995,673	6,113,458
	Friedman	8.300	5.433	3.067	9.933	4.200	6.400	8.500	6.167	1.500	1.500
25-bar	average	4.08E + 08	5.33E + 08	5.49E + 08	3.26E + 08	5.34E + 08	5.15E + 08	4.7E + 08	5.43E + 08	5.59E + 08	5.6E + 08
	max	4.92E + 08	5.55E + 08	5.53E + 08	4.99E + 08	5.45E + 08	5.38E + 08	5.15E + 08	5.59E + 08	**5.63E + 08**	5.61E + 08
	min	2.71E + 08	4.75E + 08	5.43E + 08	0	5.2E + 08	4.62E + 08	4.34E + 08	5.27E + 08	5.46E + 08	5.58E + 08
	std	62,773,336	17,204,512	2,075,881	1.43E + 08	7,442,694	19,129,030	16,749,685	9,303,211	3,613,499	807457.4
	Friedman	9.133	5.267	3.433	9.500	5.433	6.733	8.233	4.200	1.667	1.400
37-bar	average	1.25E + 08	1.37E + 08	1.51E + 08	1.22E + 08	1.47E + 08	1.37E + 08	1.17E + 08	1.4E + 08	1.55E + 08	1.53E + 08
	max	1.44E + 08	1.44E + 08	1.53E + 08	1.45E + 08	1.52E + 08	1.53E + 08	1.23E + 08	1.49E + 08	**1.56E + 08**	1.55E + 08
	min	74,429,861	1.27E + 08	1.5E + 08	4,645,312	1.43E + 08	43,400,281	1.08E + 08	1.27E + 08	1.54E + 08	1.5E + 08
	std	15,241,020	4,124,711	661944.7	27,273,598	2,404,063	19,006,014	3,623,926	5,679,951	428710.7	1,171,606
	Friedman	8.133	6.933	2.967	8.367	4.133	6.033	9.500	5.833	1.067	2.033
60-bar	average	3.7E + 08	3.87E + 08	4.44E + 08	3.4E + 08	4.09E + 08	4.21E + 08	3.21E + 08	3.97E + 08	4.73E + 08	4.44E + 08
	max	3.9E + 08	4.16E + 08	4.57E + 08	4.13E + 08	4.34E + 08	4.39E + 08	3.42E + 08	4.2E + 08	**4.76E + 08**	4.55E + 08
	min	3.35E + 08	3.41E + 08	4.3E + 08	27,987,312	3.73E + 08	3.64E + 08	2.95E + 08	3.72E + 08	4.66E + 08	4.2E + 08
	std	14,452,720	15,601,343	7,492,358	68,151,879	15,065,086	14,223,960	12,769,726	13,856,795	2,105,812	7,068,600
	Friedman	7.900	6.767	2.567	8.533	5.333	4.267	9.833	6.233	1.000	2.567
72-bar	average	2.63E + 09	2.79E + 09	3.13E + 09	2.54E + 09	2.98E + 09	2.82E + 09	2.26E + 09	2.88E + 09	3.2E + 09	3.08E + 09
	max	2.85E + 09	2.97E + 09	3.15E + 09	3.01E + 09	3.05E + 09	3.03E + 09	2.43E + 09	3.06E + 09	**3.21E + 09**	3.17E + 09
	min	1.97E + 09	2.62E + 09	3.1E + 09	1.67E + 08	2.89E + 09	1.7E + 09	2.04E + 09	2.58E + 09	3.18E + 09	2.97E + 09
	std	1.84E + 08	88,286,459	12,129,868	7.44E + 08	42,795,380	2.93E + 08	75,044,109	1.2E + 08	5,573,982	54,633,046
	Friedman	8.233	7.267	2.200	7.233	4.400	6.033	9.700	5.900	1.000	3.033
120-bar	average	5.99E + 10	7.91E + 10	8.19E + 10	3.35E + 10	7.99E + 10	7.42E + 10	6.81E + 10	7.69E + 10	8.43E + 10	8.45E + 10
	max	7.77E + 10	8.21E + 10	8.28E + 10	7.85E + 10	8.18E + 10	8E + 10	7.34E + 10	8.09E + 10	**8.48E + 10**	8.47E + 10
	min	3.46E + 10	7.11E + 10	8.1E + 10	0	7.82E + 10	1.61E + 10	6.33E + 10	7.13E + 10	8.33E + 10	8.42E + 10
	std	1.13E + 10	2.38E + 09	3.7E + 08	2.43E + 10	9.28E + 08	1.14E + 10	2.76E + 09	2.24E + 09	3.48E + 08	1.14E + 08
	Friedman	8.767	4.933	3.067	9.667	4.600	6.533	8.267	6.167	1.667	1.333
200-bar	average	2.51E + 10	2.34E + 10	2.85E + 10	2.09E + 10	2.68E + 10	2.64E + 10	1.99E + 10	2.72E + 10	2.94E + 10	2.85E + 10
	max	2.72E + 10	2.59E + 10	2.88E + 10	2.64E + 10	2.75E + 10	2.74E + 10	2.16E + 10	2.83E + 10	**2.94E + 10**	2.91E + 10
	min	2.05E + 10	2.22E + 10	2.83E + 10	1.04E + 10	2.57E + 10	2.37E + 10	1.86E + 10	2.53E + 10	2.93E + 10	2.74E + 10
	std	1.6E + 09	7.71E + 08	1.37E + 08	3.58E + 09	4.42E + 08	8.84E + 08	7.27E + 08	6.85E + 08	20,332,826	3.7E + 08
	Friedman	7.067	8.067	2.433	8.900	5.233	5.633	9.667	4.400	1.000	2.600
942-bar	average	1.8E + 14	1.55E + 14	1.97E + 14	1.89E + 14	1.81E + 14	1.87E + 14	1.32E + 14	1.84E + 14	2.1E + 14	1.97E + 14
	max	1.89E + 14	1.65E + 14	1.99E + 14	2E + 14	1.87E + 14	2.01E + 14	1.4E + 14	1.93E + 14	**2.1E + 14**	2.04E + 14
	min	1.55E + 14	1.41E + 14	1.94E + 14	1.67E + 14	1.76E + 14	1.54E + 14	1.28E + 14	1.74E + 14	2.09E + 14	1.91E + 14
	std	8.25E + 12	5.98E + 12	1.33E + 12	7.84E + 12	2.8E + 12	1.02E + 13	3.31E + 12	4.4E + 12	2.74E + 11	3.41E + 12
	Frank	6.467	9.000	2.867	4.633	7.100	5.133	10.000	6.100	1.000	2.700
Average Friedman		8.000	6.708	2.825	8.346	5.054	5.846	9.213	5.625	1.238	2.146
Overall Friedman rank		8	7	3	9	4	6	10	5	1	2

**Table 3:** The generational distance (GD) metric of the considered truss structures.

GD		MOALO	MODA	MOMVO	MOWCA	MSSA	NSGA-II	DEMO	MOBA	MOCRY2 A	MOCRY
10-bar	average	82.7131	87.59163	70.47981	22.25105	102.9799	86.96031	263.2339	63.40685	64.29878	54.38849
	max	119.3397	144.2261	97.65523	63.64834	208.0006	147.1875	334.439	124.9234	71.08283	65.8335
	min	38.66082	52.93379	47.77595	0	57.97036	52.3558	204.2038	38.17118	59.21368	45.42797
	std	18.88218	21.73929	13.35028	14.66381	38.25931	23.44446	33.98838	20.99984	3.379615	4.817122
	Friedman	6.333	6.667	5.267	1.167	7.400	6.700	10.000	4.100	4.567	2.800
25-bar	average	33.79893	53.56172	32.56686	31.33904	74.24278	38.25654	105.0261	26.51217	21.75229	27.26952
	max	96.1072	74.45301	50.27021	162.4908	111.4386	61.07276	143.5362	36.25331	24.68517	35.29048
	min	12.45335	30.46007	25.1618	0	36.69979	19.19497	61.42248	18.35706	18.50279	20.55782
	std	16.14679	11.56095	6.225057	31.55113	19.02154	9.924137	20.15427	4.213317	1.333073	3.431376
	Friedman	4.667	7.700	5.167	3.733	8.900	5.833	9.800	3.533	1.900	3.767
37-bar	average	17.32447	32.88394	14.84693	11.17736	25.96413	25.39695	73.72979	26.50876	11.60565	16.44128
	max	26.51095	44.42958	20.94492	27.08912	52.86685	33.85097	127.366	35.30699	13.31615	29.01149
	min	12.12212	19.50391	10.06001	3.5325	13.47319	12.95461	37.49198	20.56015	10.34733	9.78902
	std	3.658182	6.797258	2.528716	5.257875	9.152806	5.415314	25.77997	4.365317	0.655056	4.110909
	Friedman	4.567	8.133	3.467	2.000	6.967	6.800	9.967	7.133	1.833	4.133
60-bar	average	43.14495	83.82058	31.94236	27.38585	58.5019	54.61234	187.102	90.49675	23.33581	66.88495
	max	65.4209	112.5462	43.17429	151.7614	84.88041	71.80981	226.557	168.1119	26.71557	120.3429
	min	30.78355	63.31996	25.32175	11.44169	37.4402	37.01083	126.52	58.90248	21.11755	47.25334
	std	8.411214	13.27017	4.295376	25.67342	12.61553	9.450814	23.20639	27.05104	1.472132	14.54626
	Friedman	4.333	8.167	2.900	1.900	5.833	5.467	10.000	8.033	1.600	6.767
72-bar	average	113.7969	234.7376	65.99277	48.06035	194.0539	121.5656	462.2427	123.9398	46.41081	113.6702
	max	183.4317	397.1979	108.5688	131.2342	273.7678	235.1241	683.9107	197.313	53.11109	196.6088
	min	64.33269	110.3297	47.91745	10.33995	119.5942	56.75353	266.5388	68.81717	41.62294	54.38655
	std	32.09438	64.54584	13.55068	28.36304	43.96003	35.63233	90.47534	34.31015	2.878633	40.20948
	Friedman	5.233	8.500	3.267	1.733	7.900	5.700	10.000	5.800	1.633	5.233
120-bar	average	566.4333	440.1025	446.7862	158.2029	609.7665	395.7652	905.7258	296.5587	438.6884	360.4737
	max	748.9397	600.1568	576.058	501.967	933.3694	576.8113	1298.245	355.8445	474.6862	396.6171
	min	346.0603	309.9338	340.298	0	407.4997	231.6233	574.8111	241.4981	408.557	330.7751
	std	105.3897	72.12498	65.42489	123.7237	129.2193	84.38627	154.9107	30.46965	18.07715	15.90806
	Friedman	7.700	5.500	6.100	1.500	8.267	4.433	9.933	2.133	5.900	3.533
200-bar	average	504.2952	1079.169	202.0283	192.4569	528.5552	458.281	1946.389	373.6981	117.5304	421.148
	max	975.138	1557.253	285.2356	1011.762	840.6578	704.8619	2644.011	600.5547	132.3248	920.977
	min	188.5615	370.3282	114.4864	78.26861	366.8544	209.2656	1222.997	202.3372	98.64228	178.6481
	std	203.0758	291.6666	49.83722	199.4698	113.6445	126.2982	369.6485	101.7148	6.960366	154.0638
	Friedman	6.133	8.800	2.867	2.167	6.933	5.900	10.000	5.100	1.667	5.433
942-bar	average	86058.33	56955.03	32728.55	30084.31	37891.12	35905.57	68258.63	57,636	12084.55	68672.54
	max	134,469	102314.4	40297.92	116011.2	72321.96	75063.23	104764.9	115118.7	14469.82	101319.7
	min	59956.43	31619.95	22398.71	17926.01	19731.02	16425.43	42376.25	37133.42	9476.139	34707.09
	std	15309.76	18582.65	4312.804	18688.92	13892.61	11719.56	16710.12	17998.39	1124.786	16831.67
	Frank	9.367	6.800	3.733	3.000	4.267	4.167	7.800	6.867	1.000	8.000
Average Friedman		6.042	7.533	4.096	2.150	7.058	5.625	9.688	5.338	2.513	4.958
Overall Friedman rank		7	9	3	1	8	6	10	5	2	4

**Table 4:** The inverted generational distance (IGD) metric of the considered truss structures.

IGD		MOALO	MODA	MOMVO	MOWCA	MSSA	NSGA-II	DEMO	MOBA	MOCRY2 A	MOCRY
10-bar	average	2744.358	2730.352	370.7354	6961.392	1214.04	702.6752	2976.37	2765.809	187.7996	290.6504
	max	6931.381	4530.439	665.0549	7767.213	3101.79	1881.333	4225.918	4124.009	275.5526	635.8286
	min	555.1642	1332.01	302.9059	3308.406	434.176	304.4011	1499.589	1557.788	116.4364	120.9599
	std	1489.442	874.0142	65.21475	1082.834	572.4337	356.9155	655.951	675.6004	44.36289	133.9163
	Friedman	7.200	7.133	2.800	9.967	5.233	4.033	7.767	7.533	1.267	2.067
25-bar	average	1072.365	618.6892	125.2119	1824.829	270.8738	215.3233	864.3381	409.8013	147.3373	52.45578
	max	2065.221	1586.182	167.7059	2516.887	878.8984	335.7962	1598.996	1049.033	287.8849	100.5114
	min	300.6858	101.9471	91.34614	956.0153	161.154	110.3097	429.7239	84.39536	80.30067	37.42533
	std	494.0962	382.3997	18.79522	455.4833	145.1585	65.69537	342.0581	223.1668	45.15699	13.46233
	Friedman	8.467	6.933	2.533	9.833	4.933	4.333	7.933	5.933	3.100	1.000
37-bar	average	466.9905	539.2546	109.7838	850.437	331.3302	197.1149	616.4427	466.9954	91.51411	98.47713
	max	1166.69	877.8927	172.7865	1389.121	570.0807	568.6185	900.3673	841.8574	141.4256	265.3774
	min	192.4607	178.1916	66.23636	495.3954	155.1149	56.77685	361.4781	212.4002	42.11174	32.38007
	std	218.1968	171.3273	24.48297	209.9577	109.5864	104.6836	164.5285	140.4578	26.12303	47.05607
	Friedman	6.733	7.467	2.533	9.733	5.567	3.800	8.267	6.833	1.933	2.133
60-bar	average	1762.582	1189.572	369.6954	2085.805	1331.702	603.7896	1365.876	1216.377	234.6057	189.9643
	max	2163.957	2120.893	534.2461	3124.152	1966.718	1054.247	1772.853	1732.629	387.7638	571.686
	min	1372.709	485.514	189.1424	1391.642	642.9563	238.5766	868.1966	889.187	64.05481	73.62395
	std	194.9818	425.2922	89.9813	405.3676	361.504	227.0562	229.767	203.9372	83.93164	105.6752
	Friedman	8.800	6.500	3.100	9.333	6.867	3.800	7.067	6.333	1.733	1.467
72-bar	average	1671.779	1982.489	531.9122	3178.688	1204.654	705.4993	2816.354	2058.69	281.8292	1091.045
	max	3444.548	3120.34	669.802	5456.157	2568.598	1965.585	3904.449	3550.061	864.9589	2292.418
	min	783.7408	963.5836	336.5824	1290.591	480.42	330.7954	1971.18	842.7337	119.2682	324.398
	std	542.8913	611.9677	70.32905	1001.697	677.6934	361.5506	519.872	667.9315	149.8946	500.5578
	Friedman	6.300	7.233	2.400	9.267	4.833	3.200	8.967	6.900	1.200	4.700
120-bar	average	26448.1	15978.95	2430.496	46176.96	9061.295	6095.773	24663.64	19914.3	1393.466	2316.573
	max	53476.48	35163.48	3639.952	58037.57	19658.88	24885.19	35148.36	31462.48	2705.278	4883.482
	min	8151.234	4350.534	1883.099	10075.61	4921.344	2025.021	14941.02	7418.649	665.8734	1063.466
	std	12465.59	6587.395	437.166	12512.62	3806.574	4487.697	5150.295	4667.766	469.5698	1050.035
	Friedman	8.033	6.467	2.567	9.667	5.033	4.333	8.067	7.267	1.233	2.333
200-bar	average	4641.748	6843.539	3053.324	9355.436	5983.023	3325.558	7046.48	5058.751	1235.321	1028.825
	max	6925.92	8945.574	4146.575	11846.7	7406.105	5439.907	8646.937	7088.337	1948.653	1744.178
	min	2979.263	4568.283	1522.721	6384.765	4049.683	1339.217	5428.189	3541.39	817.1641	539.7179
	std	1171.38	1173.361	631.7064	1224.396	904.7937	856.9758	742.695	848.531	262.0008	347.1538
	Friedman	5.400	8.100	3.533	9.700	6.967	3.833	8.367	6.100	1.733	1.267
942-bar	average	568951.9	628659.1	502202.6	489260.9	467396.3	205191.3	769642.2	420366.4	185484.7	80153.4
	max	1,003,714	764264.6	791105.7	1,035,919	657278.4	482,036	823017.5	596,232	321,115	116669.6
	min	210611.9	525717.1	372563.1	271,556	372468.5	130453.6	660737.4	284844.5	105739.9	50446.25
	std	205,822	61108.45	93434.22	147504.9	62041.23	69499.03	39174.65	73458.63	54235.26	20338.69
	Frank	7.367	8.333	6.400	6.200	5.767	2.800	9.733	5.067	2.333	1.000
Average Friedman		7.288	7.271	3.233	9.213	5.650	3.767	8.271	6.496	1.817	1.996
Overall Friedman rank		8	7	3	10	5	4	9	6	1	2

**Table 5:** The Spacing - To- extent (STE) metric values obtained for the truss problems.

STE		MOALO	MODA	MOMVO	MOWCA	MSSA	NSGA-II	DEMO	MOBA	MOCRY2 A	MOCRY
10-bar	average	0.020809	0.018669	0.009111	0.051763	0.008267	0.014964	0.029956	0.018337	0.005532	0.008404
	max	0.033979	0.051609	0.014763	0.1	0.025432	0.037686	0.054524	0.049681	0.006796	0.013253
	min	0	0.005336	0.005495	0	0.001879	0.007133	0.009302	0.005035	0.004643	0.005848
	std	0.009169	0.012814	0.002213	0.040742	0.005792	0.005407	0.01282	0.01032	0.000539	0.001776
	Friedman	7.033	6.400	4.067	7.400	3.200	6.433	8.467	6.433	1.800	3.767
25-bar	average	0.020259	0.016676	0.008119	0.033849	0.009753	0.014816	0.018139	0.016143	0.005119	0.007483
	max	0.034885	0.05081	0.01361	0.1	0.018962	0.029527	0.052892	0.031566	0.00601	0.012279
	min	0	0.00568	0.003444	0	0.003625	0.008305	0.008973	0.007695	0.004484	0.005012
	std	0.00837	0.010468	0.002588	0.024506	0.004262	0.004858	0.008615	0.006388	0.000439	0.001511
	Friedman	7.700	6.467	3.400	8.400	4.100	6.500	7.367	6.733	1.400	2.933
37-bar	average	0.020991	0.0172	0.008933	0.022961	0.008412	0.014127	0.02089	0.011604	0.006201	0.007572
	max	0.032439	0.05944	0.013925	0.048603	0.033887	0.031732	0.038693	0.028485	0.008455	0.012487
	min	0.003899	0.00567	0.005734	0	0.002881	0.000965	0.007602	0.004997	0.004614	0.00523
	std	0.007471	0.01289	0.001703	0.012533	0.005893	0.006437	0.007924	0.00571	0.000978	0.001403
	Friedman	8.300	6.267	4.333	8.033	3.300	6.267	8.033	5.133	2.000	3.333
60-bar	average	0.027057	0.01987	0.008803	0.020327	0.008559	0.012721	0.033298	0.011054	0.005639	0.008792
	max	0.045569	0.045381	0.012664	0.073296	0.023047	0.024923	0.059218	0.027904	0.007639	0.012077
	min	0	0.005361	0.004696	0	0.003912	0.005097	0.013349	0.005549	0.004043	0.005363
	std	0.013076	0.011645	0.001902	0.015485	0.004683	0.004319	0.012276	0.00544	0.000924	0.001589
	Friedman	7.967	6.700	4.467	6.800	3.233	6.000	9.033	5.100	1.667	4.033
72-bar	average	0.018453	0.014414	0.009108	0.022816	0.010175	0.01569	0.028996	0.011782	0.005321	0.008869
	max	0.032673	0.046202	0.015139	0.048061	0.031563	0.028129	0.052959	0.02305	0.009052	0.017778
	min	0	0.004682	0.004493	0.006513	0.003163	0.004148	0.012183	0.004409	0.003677	0.005183
	std	0.009572	0.009029	0.002605	0.009959	0.006932	0.006166	0.010736	0.004935	0.001114	0.002984
	Friedman	6.700	5.700	4.000	8.300	3.800	6.700	9.067	5.133	1.800	3.800
120-bar	average	0.019445	0.015131	0.008771	0.040425	0.011435	0.013901	0.027635	0.016096	0.005293	0.009223
	max	0.036795	0.032996	0.015161	0.099394	0.041673	0.024686	0.067413	0.033283	0.006344	0.012546
	min	0	0.005727	0.004654	0	0.001964	0.002052	0.013646	0.006159	0.004395	0.00546
	std	0.012656	0.007003	0.002076	0.032281	0.008047	0.004871	0.012557	0.006473	0.000496	0.00152
	Friedman	6.683	6.000	3.667	7.850	4.567	5.867	8.467	6.233	1.633	4.033
200-bar	average	0.019023	0.020412	0.008898	0.027136	0.007728	0.012643	0.028385	0.008866	0.004986	0.006997
	max	0.034847	0.048721	0.012112	0.093322	0.016703	0.018835	0.047233	0.028255	0.008082	0.014419
	min	0	0.008042	0.004093	0	0.000799	0.008252	0.016331	0.004432	0.00404	0.00465
	std	0.009364	0.010497	0.001871	0.027114	0.003645	0.003313	0.008837	0.004919	0.00072	0.002126
	Friedman	7.2	7.666667	4.833333	6.933333	3.533333	6.5	9.166667	4.266667	1.6	3.3
942-bar	average	0.03013	0.016377	0.008005	0.014792	0.008876	0.01057	0.017724	0.00985	0.005559	0.005162
	max	0.043191	0.044962	0.012651	0.052419	0.021353	0.025773	0.025382	0.01696	0.006244	0.006377
	min	0.004861	0.005804	0.004858	0.006559	0.003388	0.002339	0.010652	0.004933	0.004753	0.003825
	std	0.011734	0.010282	0.001987	0.011609	0.004534	0.005227	0.003689	0.003072	0.000375	0.000676
	Frank	9.200	7.333	4.400	6.433	4.267	5.500	8.367	5.467	2.233	1.800
Average Friedman		7.598	6.567	4.146	7.519	3.750	6.221	8.496	5.563	1.767	3.375
Overall Friedman rank		9	7	4	8	3	6	10	5	1	2

**Table 6:** The overall Friedman rank obtained for the truss problems.

	MOALO	MODA	MOMVO	MOWCA	MSSA	NSGA-II	DEMO	MOBA	MOCRY2 A	MOCRY
10-bar	7.2167	6.4083	3.8000	7.1167	5.0083	5.8917	8.6833	6.0583	2.2833	2.5333
25-bar	7.4917	6.5917	3.6333	7.8667	5.8417	5.8500	8.3333	5.1000	2.0167	2.2750
37-bar	6.9333	7.2000	3.3250	7.0333	4.9917	5.7250	8.9417	6.2333	1.7083	2.9083
60-bar	7.2500	7.0333	3.2583	6.6417	5.3167	4.8833	8.9833	6.4250	1.5000	3.7083
72-bar	6.6167	7.1750	2.9667	6.6333	5.2333	5.4083	9.4333	5.9333	1.4083	4.1917
120-bar	7.7958	5.7250	3.8500	7.1708	5.6167	5.2917	8.6833	5.4500	2.6083	2.8083
200-bar	6.4500	8.1583	3.4167	6.9250	5.6667	5.4667	9.3000	4.9667	1.5000	3.1500
942-bar	8.1000	7.8667	4.3500	5.0667	5.3500	4.4000	8.9750	5.8750	1.6417	3.3750
Average Friedman	7.2318	7.0198	3.5750	6.8068	5.3781	5.3646	8.9167	5.7552	1.8333	3.1188
Overall Friedman rank	9	8	3	7	5	4	10	6	1	2

**Fig. 6:**
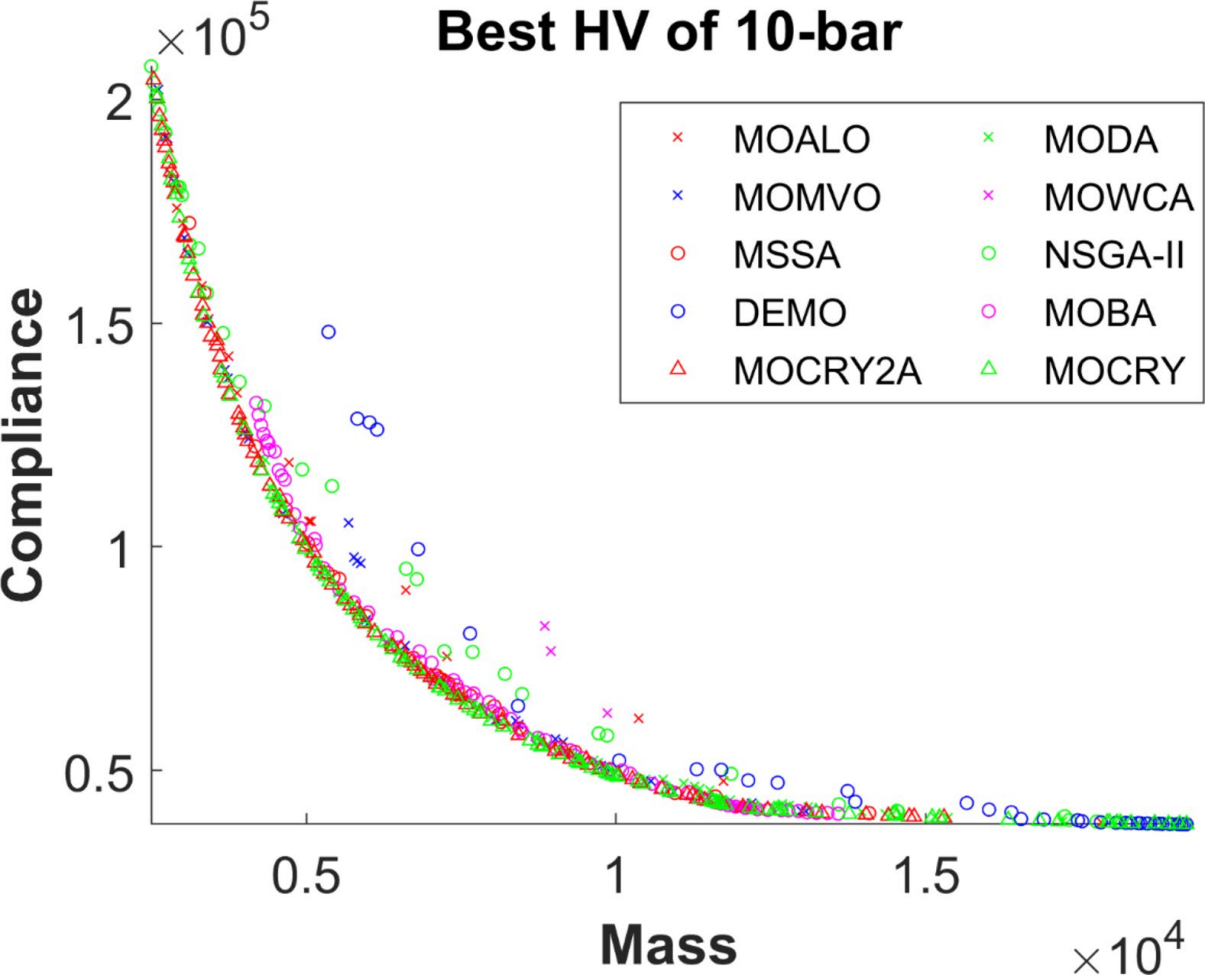
Best Pareto fronts of the 10-bar truss.

**Fig. 7:**
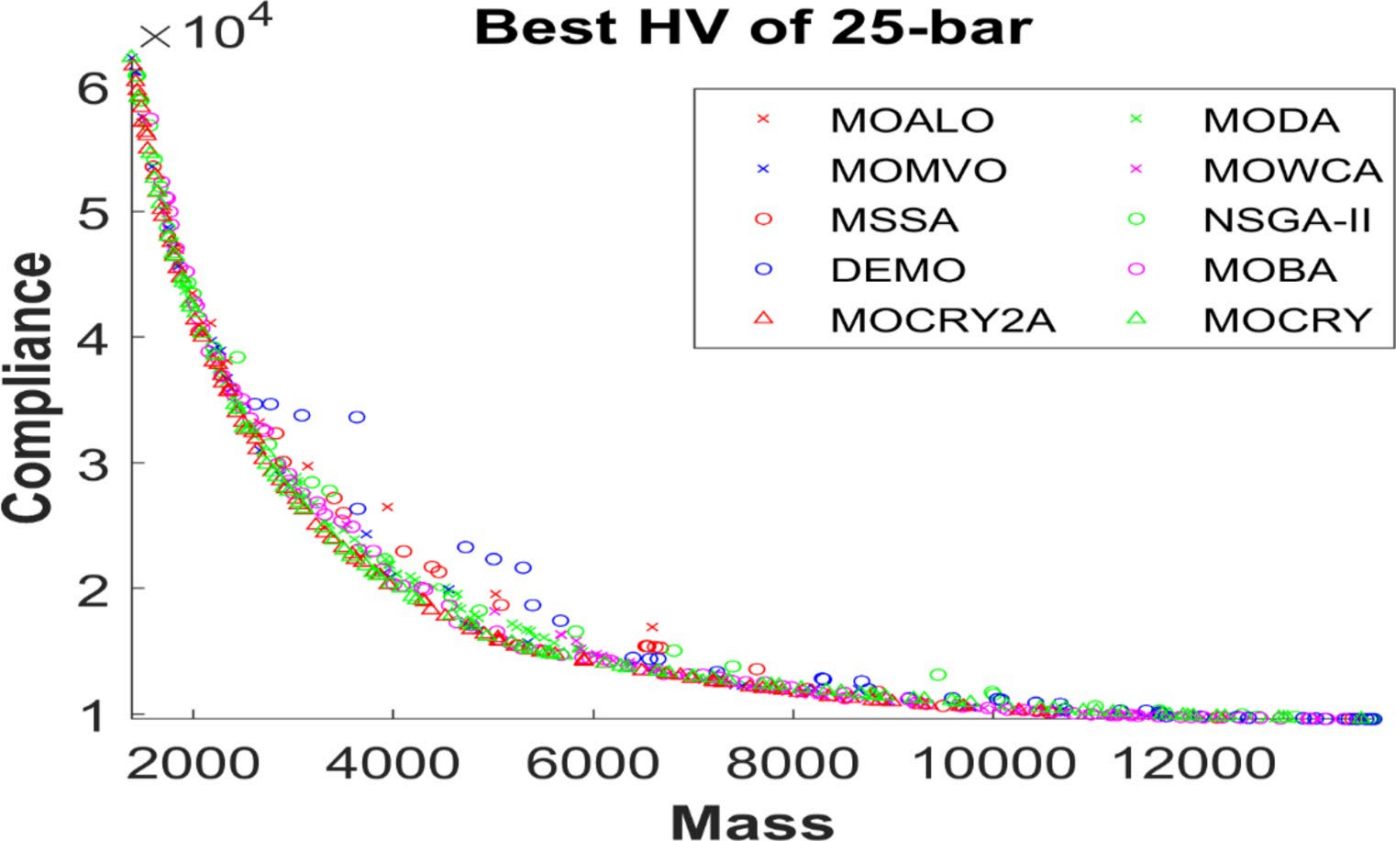
Best Pareto fronts of the 25-bar truss.

**Fig. 8:**
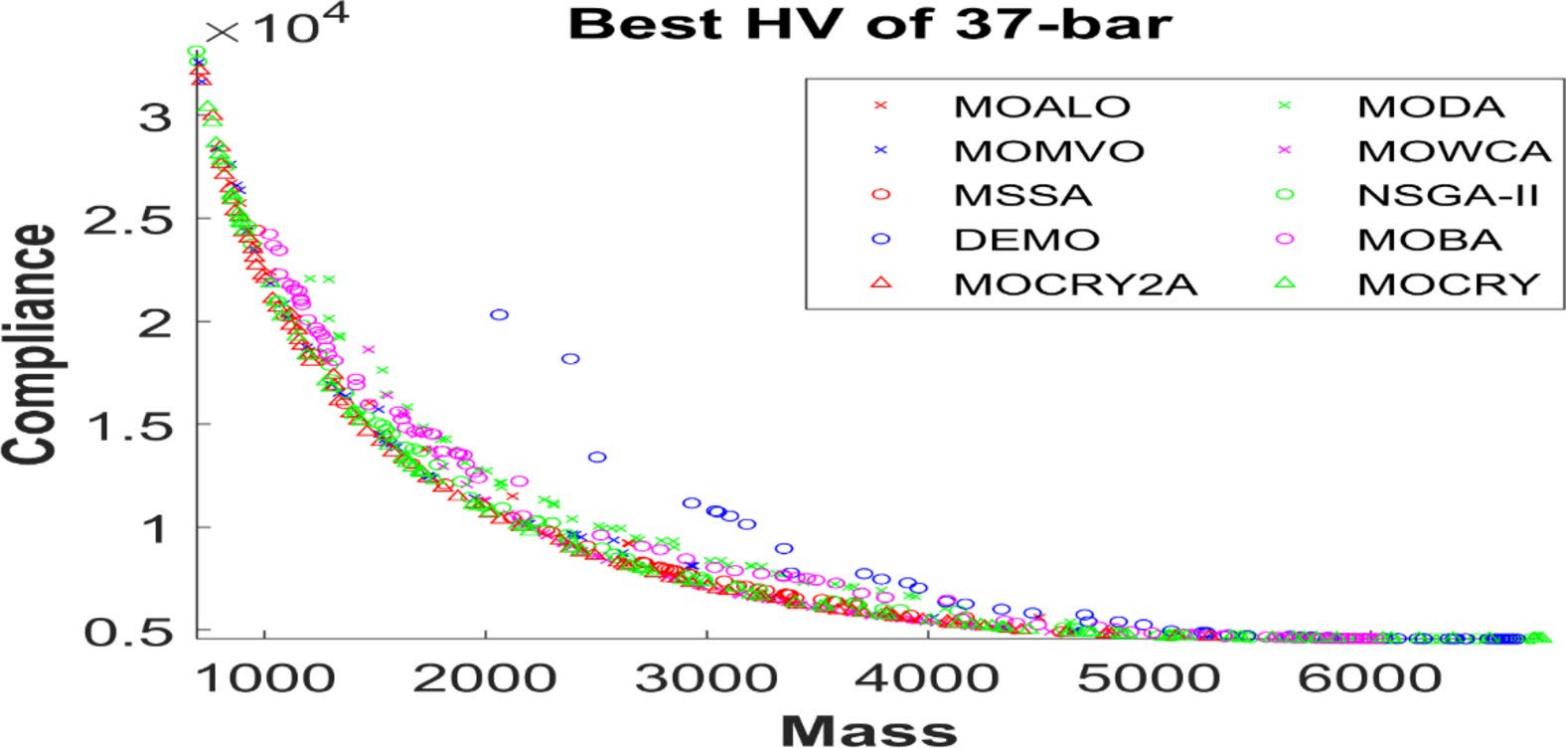
Best Pareto fronts of the 37-bar truss.

**Fig. 9:**
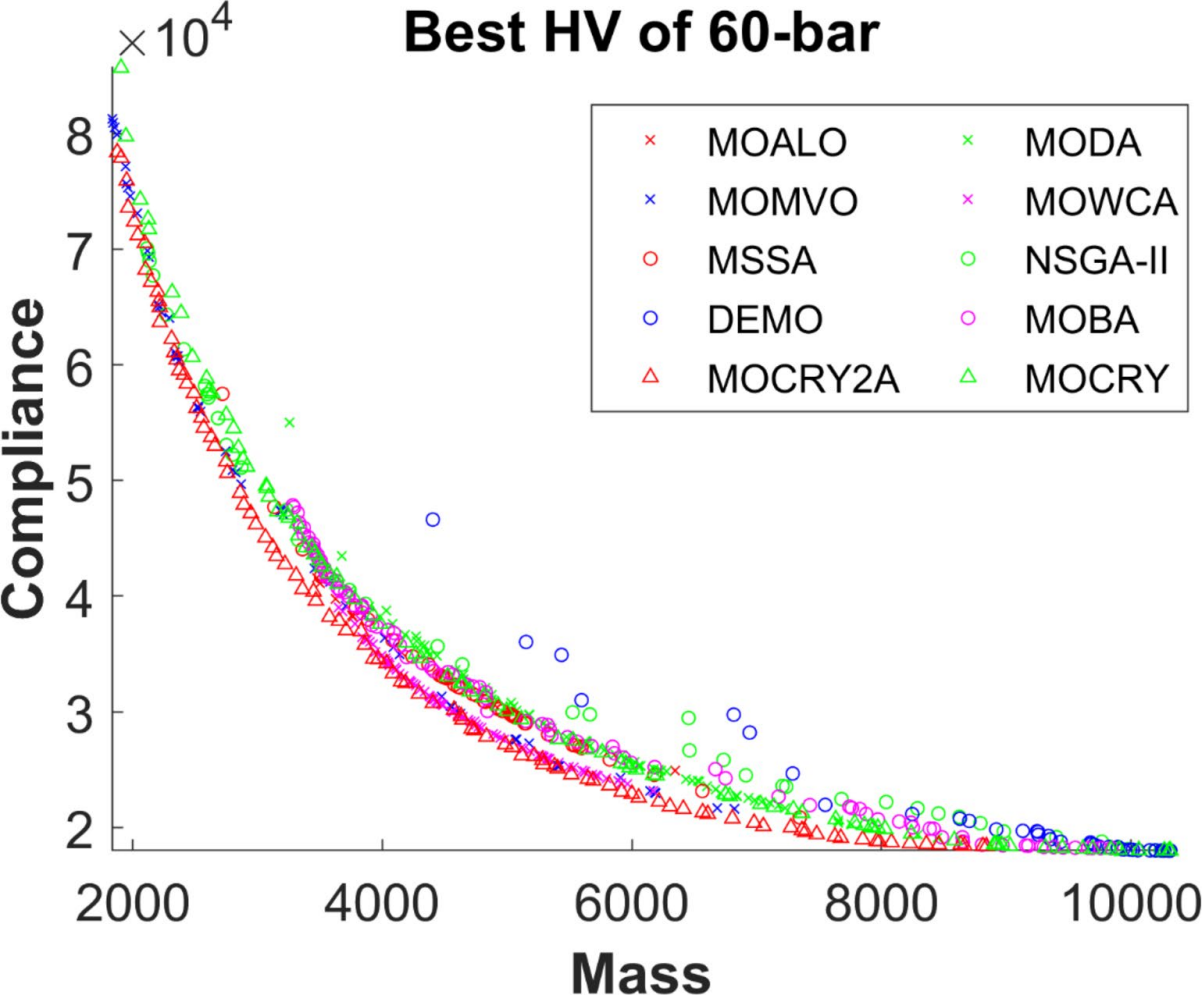
Best Pareto fronts of the 60-bar truss.

**Fig. 10:**
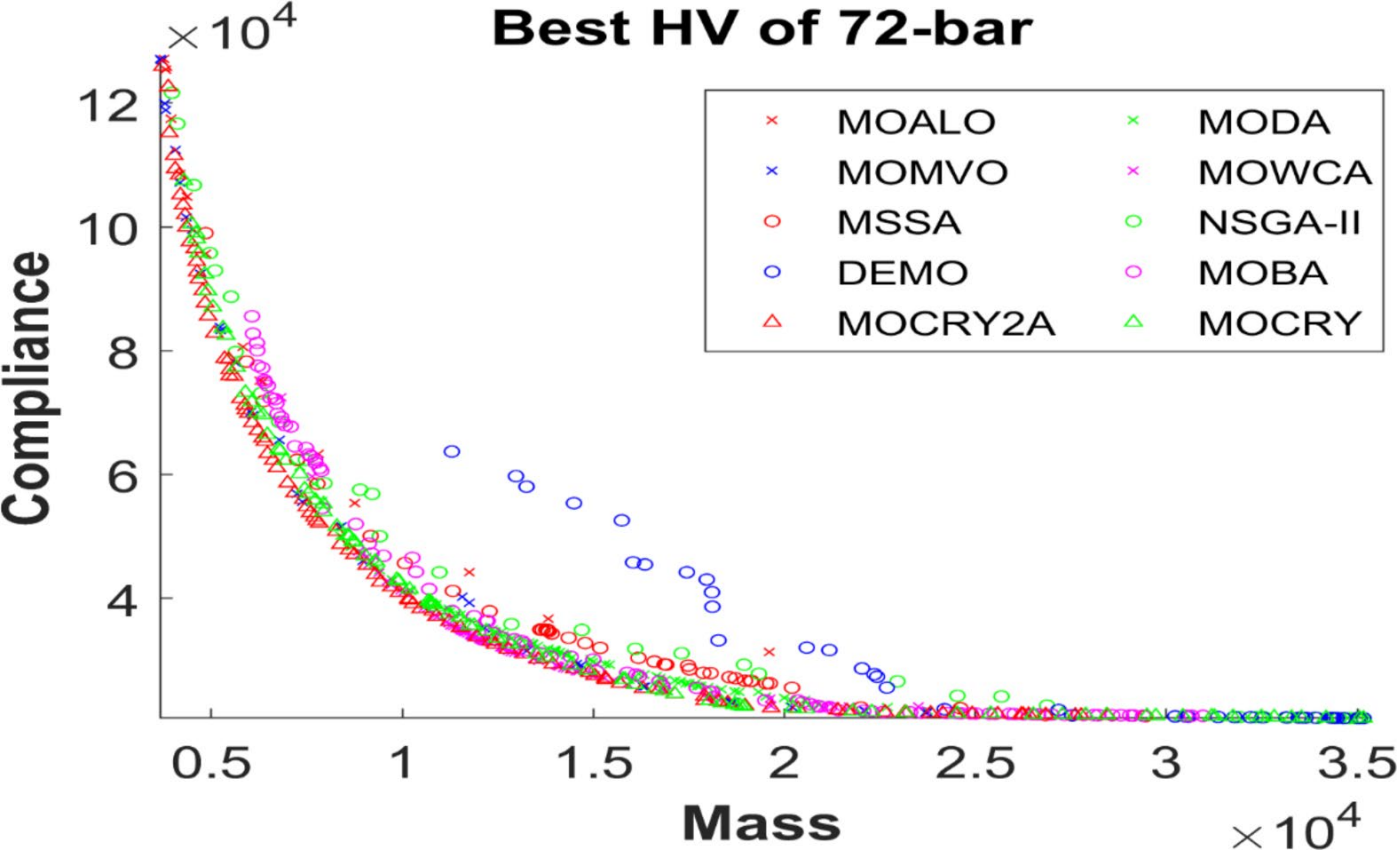
Best Pareto fronts of the 72-bar truss.

**Fig. 11:**
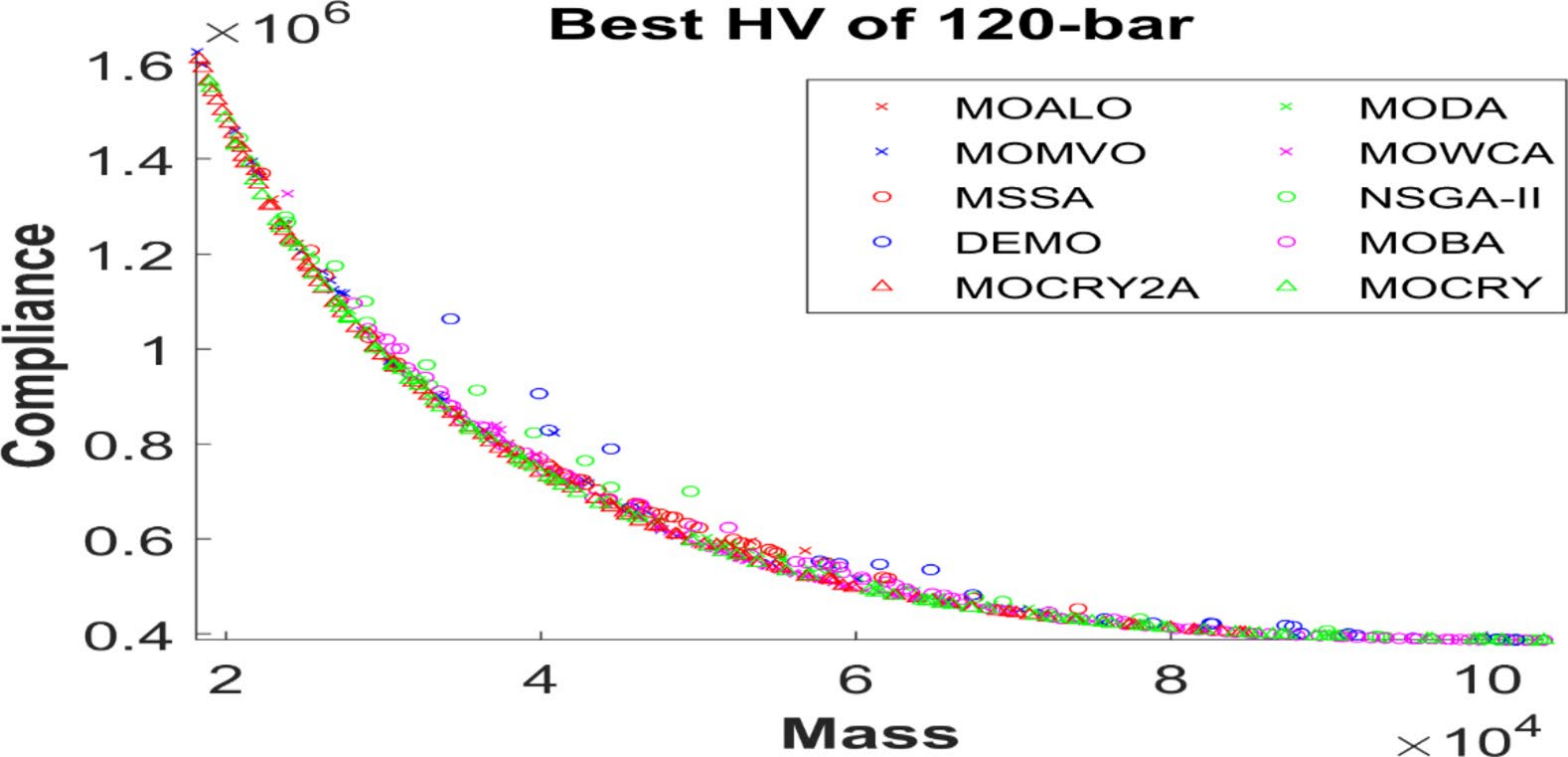
Best Pareto fronts of the 120-bar truss.

**Fig. 12:**
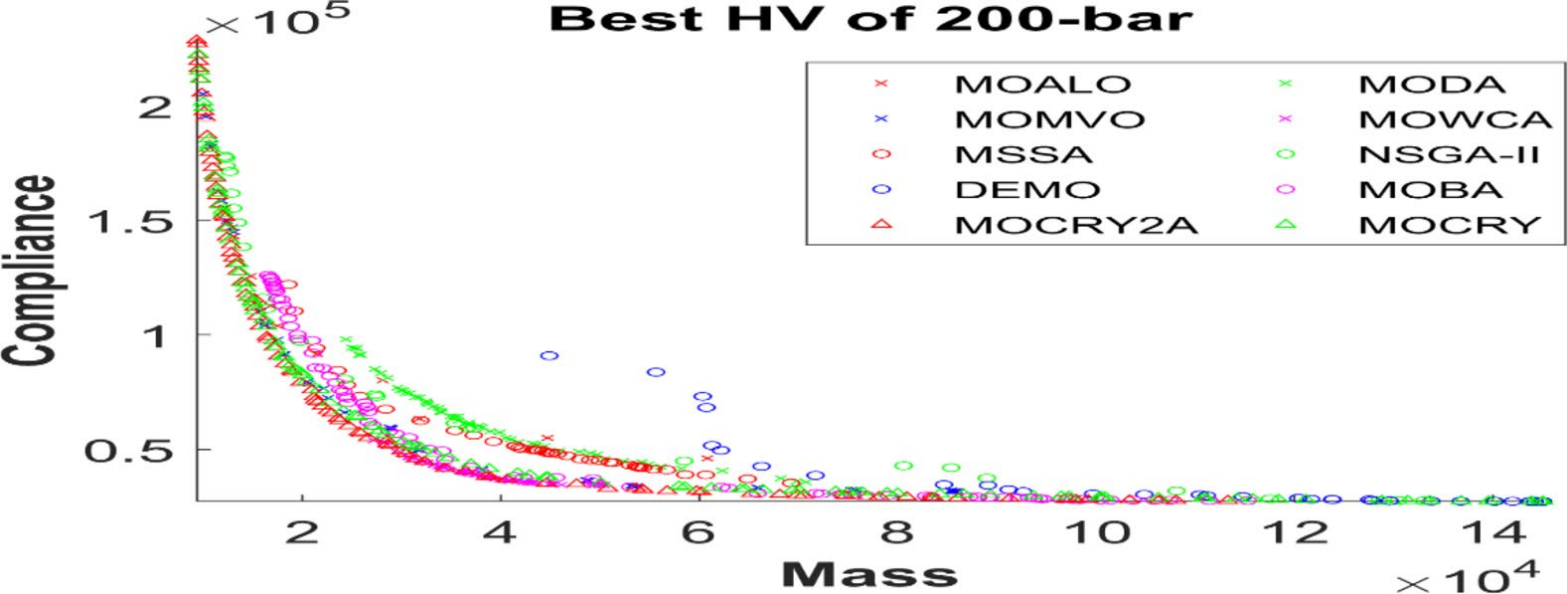
Best Pareto fronts of the 200-bar truss.

**Fig. 13:**
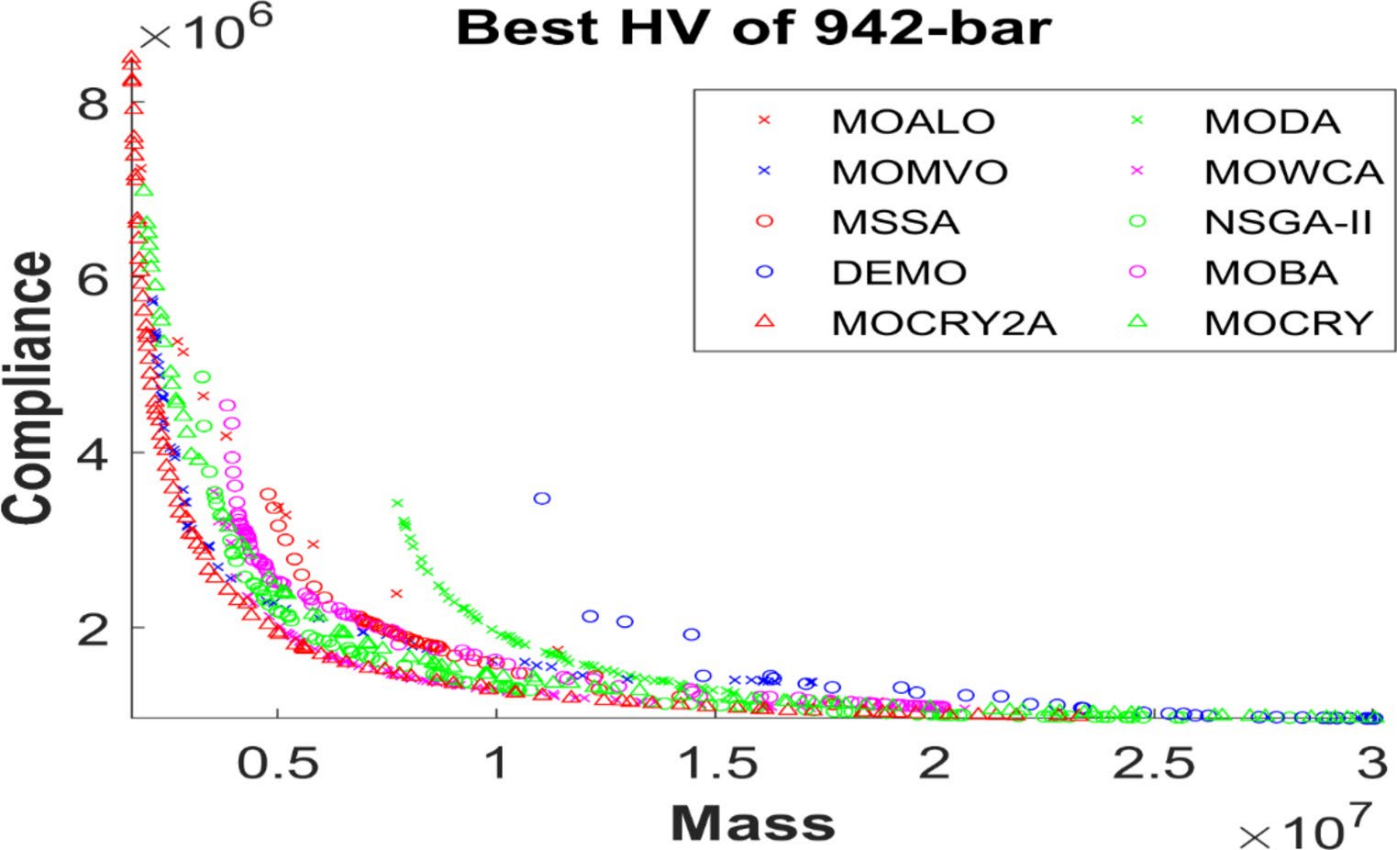
Best Pareto fronts of the 942-bar truss.

**Fig. 14:**
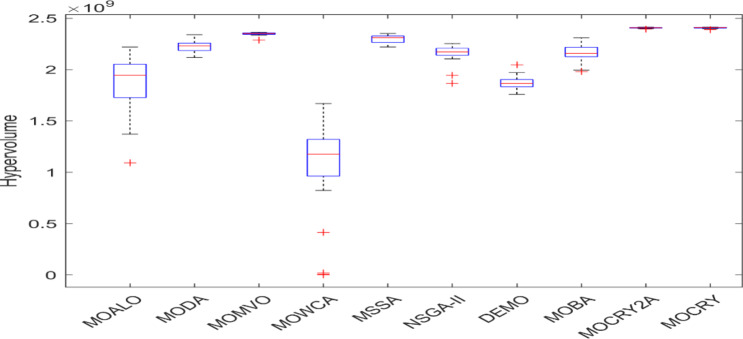
Boxplots of 10-bar truss.

**Fig. 15:**
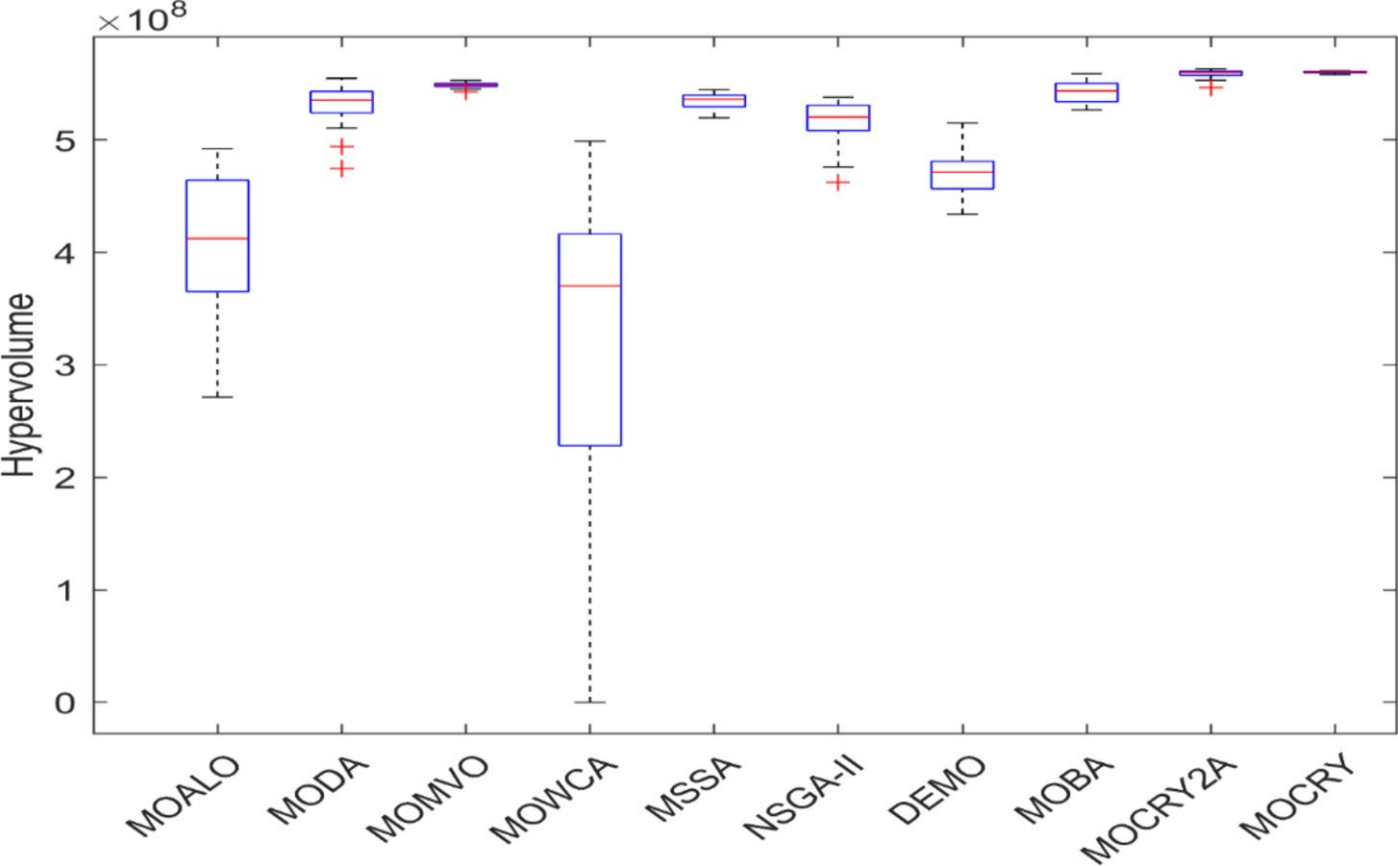
Boxplots of 25-bar truss.

**Fig. 16:**
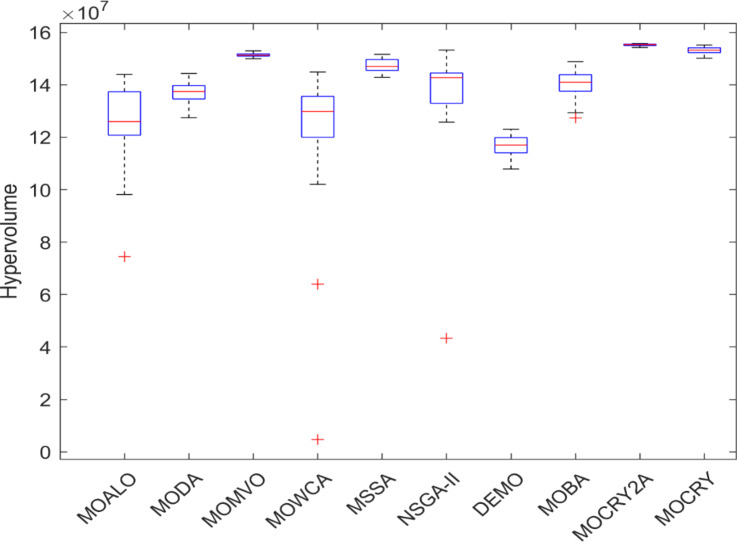
Boxplots of 37-bar truss.

**Fig. 17:**
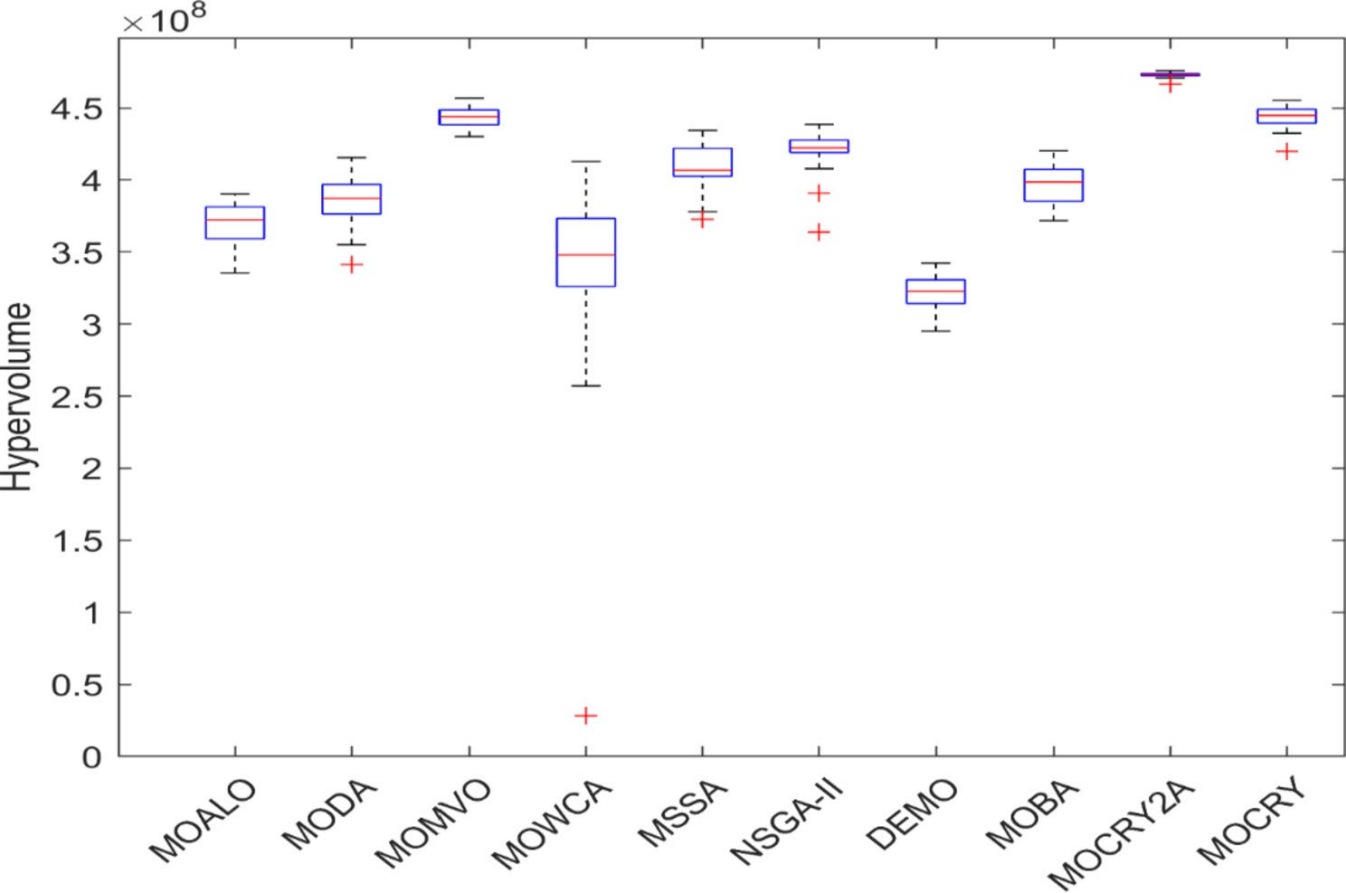
Boxplots of 60-bar truss.

**Fig. 18:**
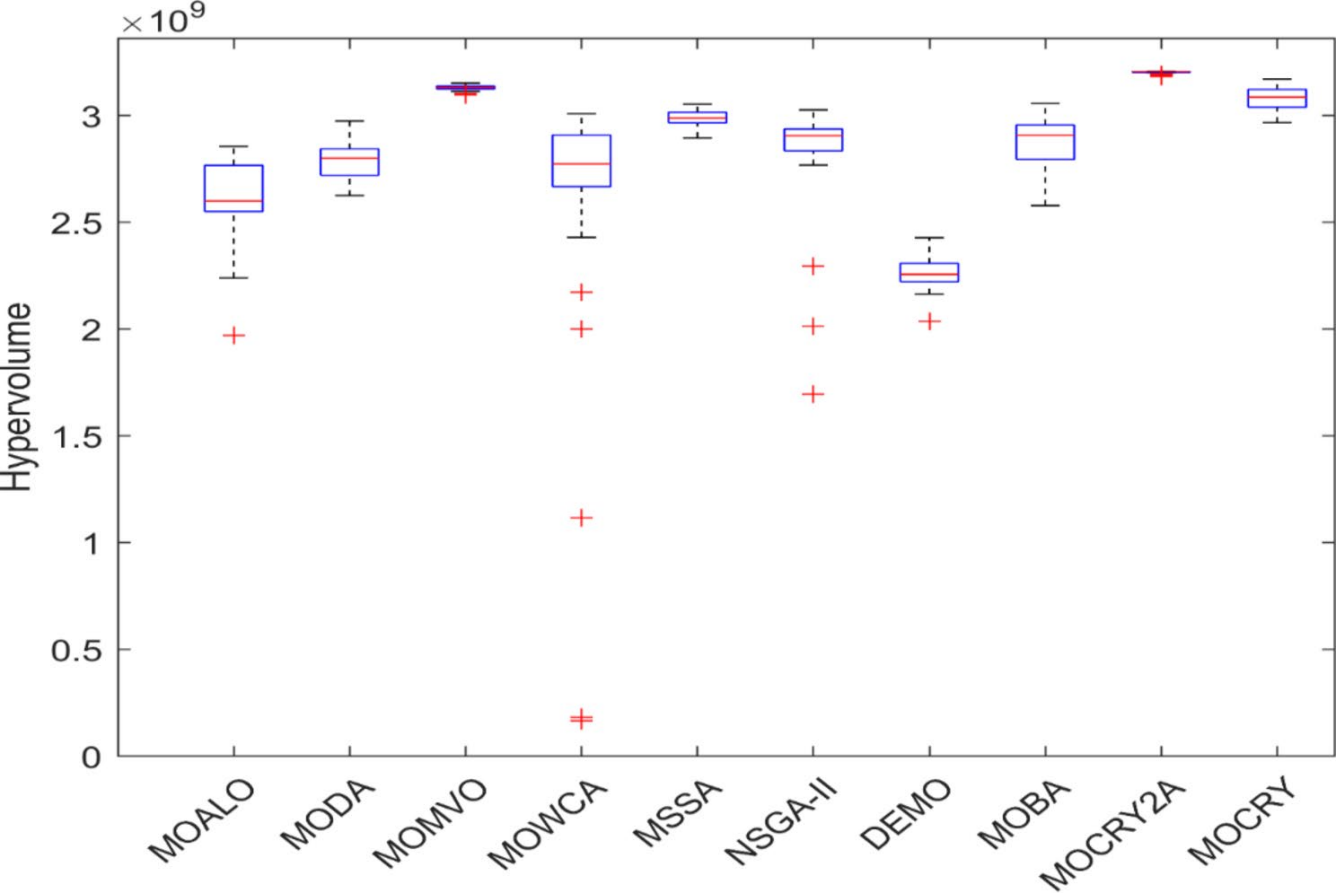
Boxplots of 72-bar truss.

**Fig. 19:**
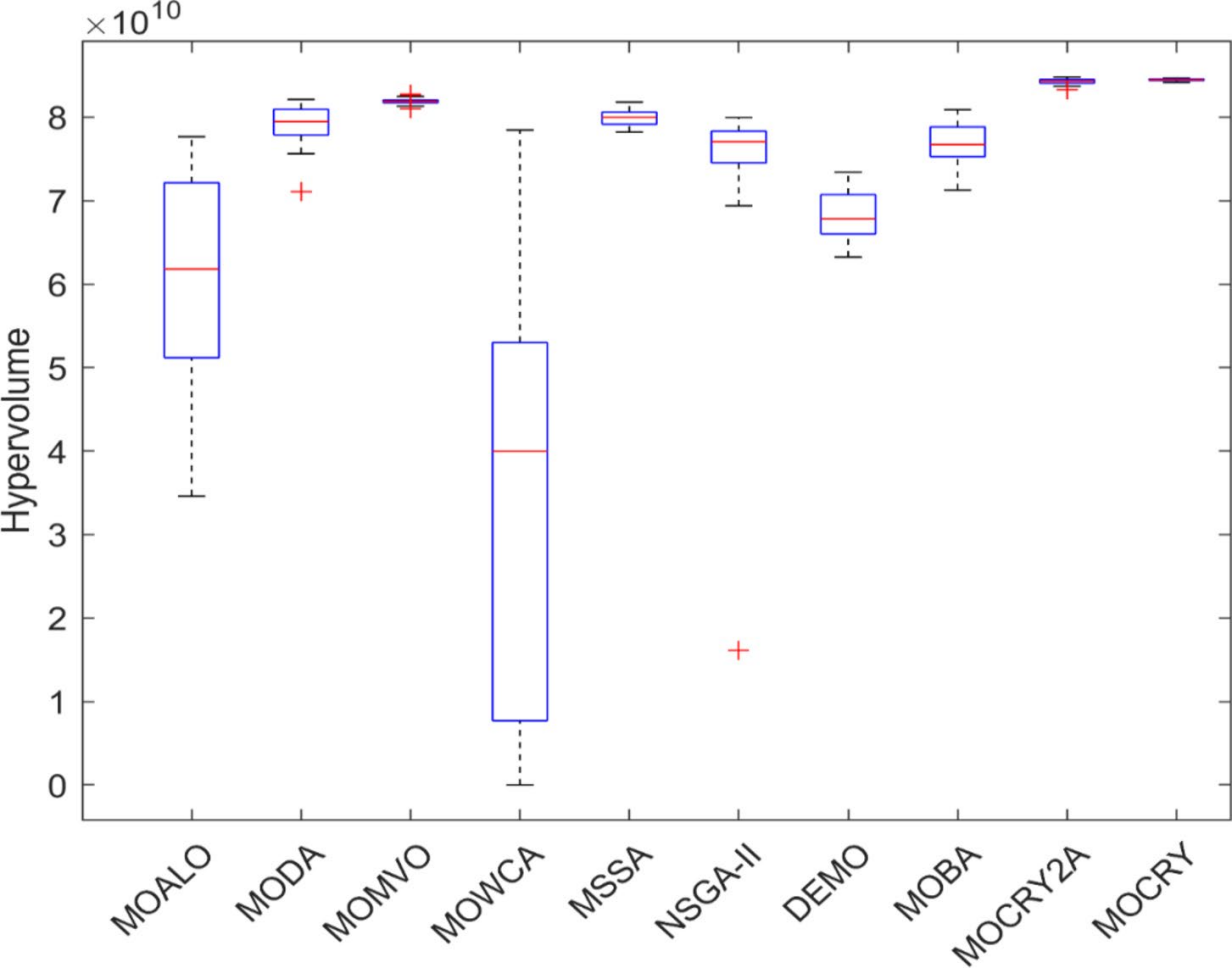
Boxplots of 120-bar truss.

**Fig. 20:**
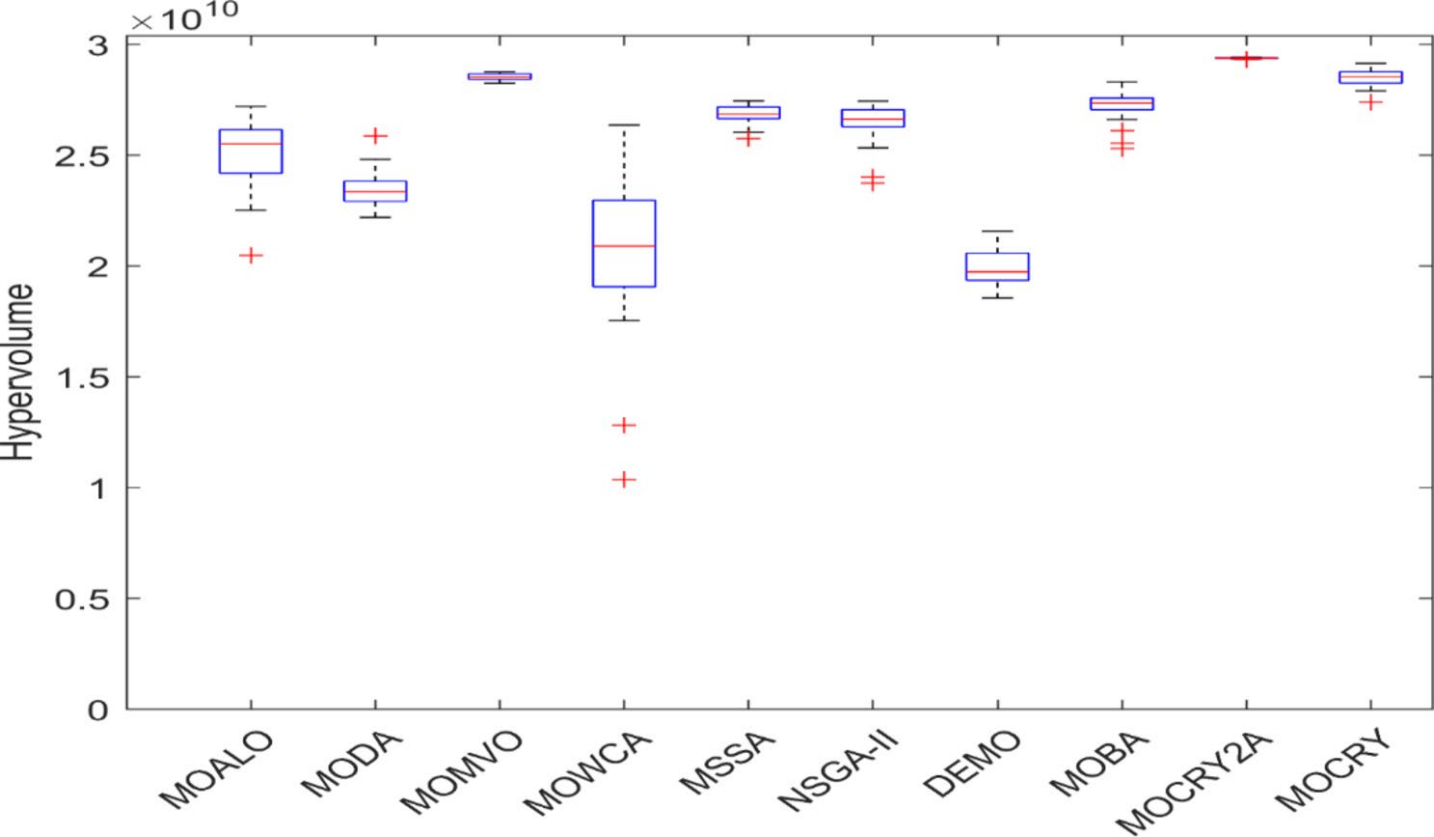
Boxplots of 200-bar truss.

**Fig. 21:**
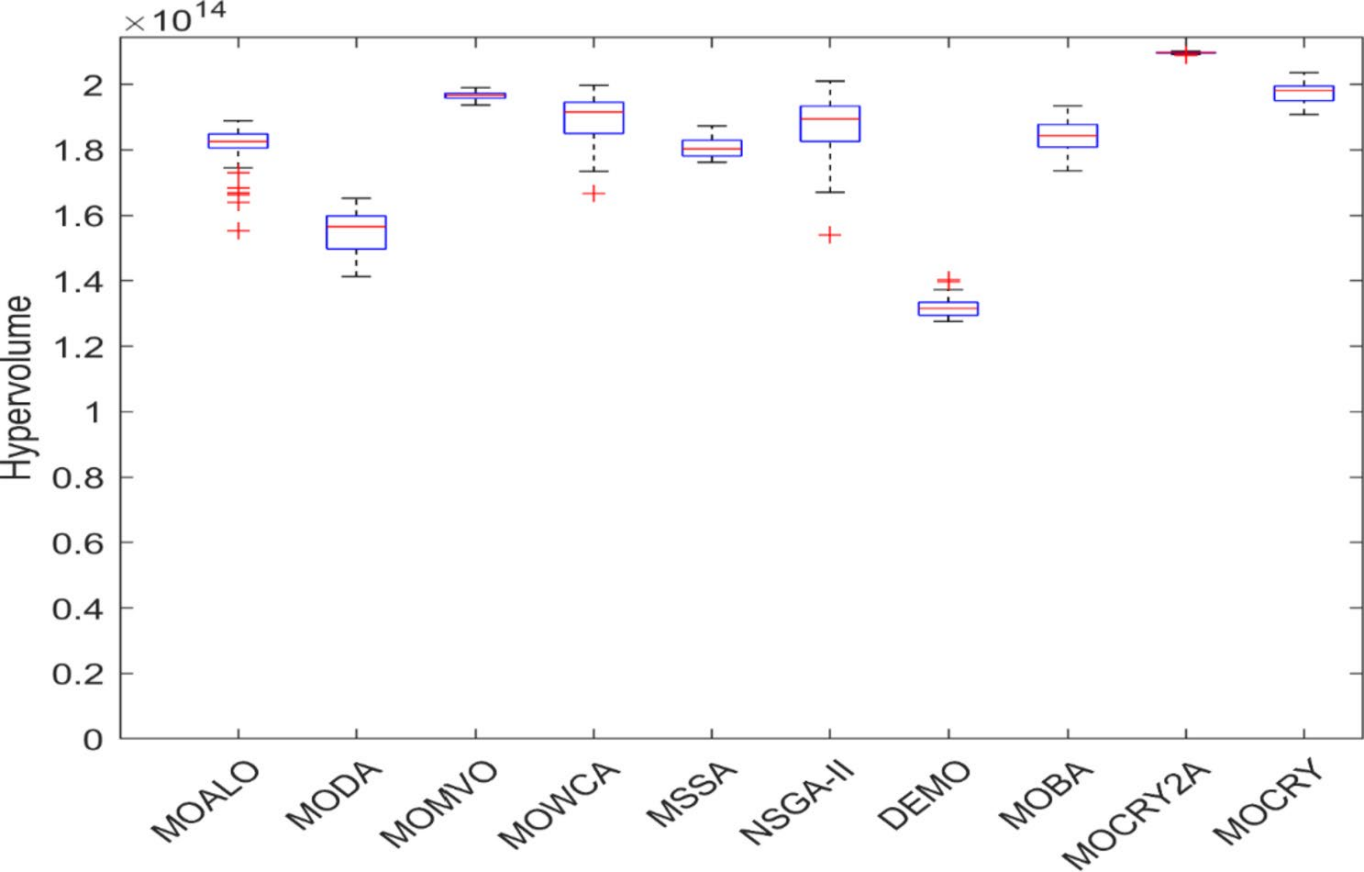
Boxplots of 942-bar truss.

**Fig. 22:**
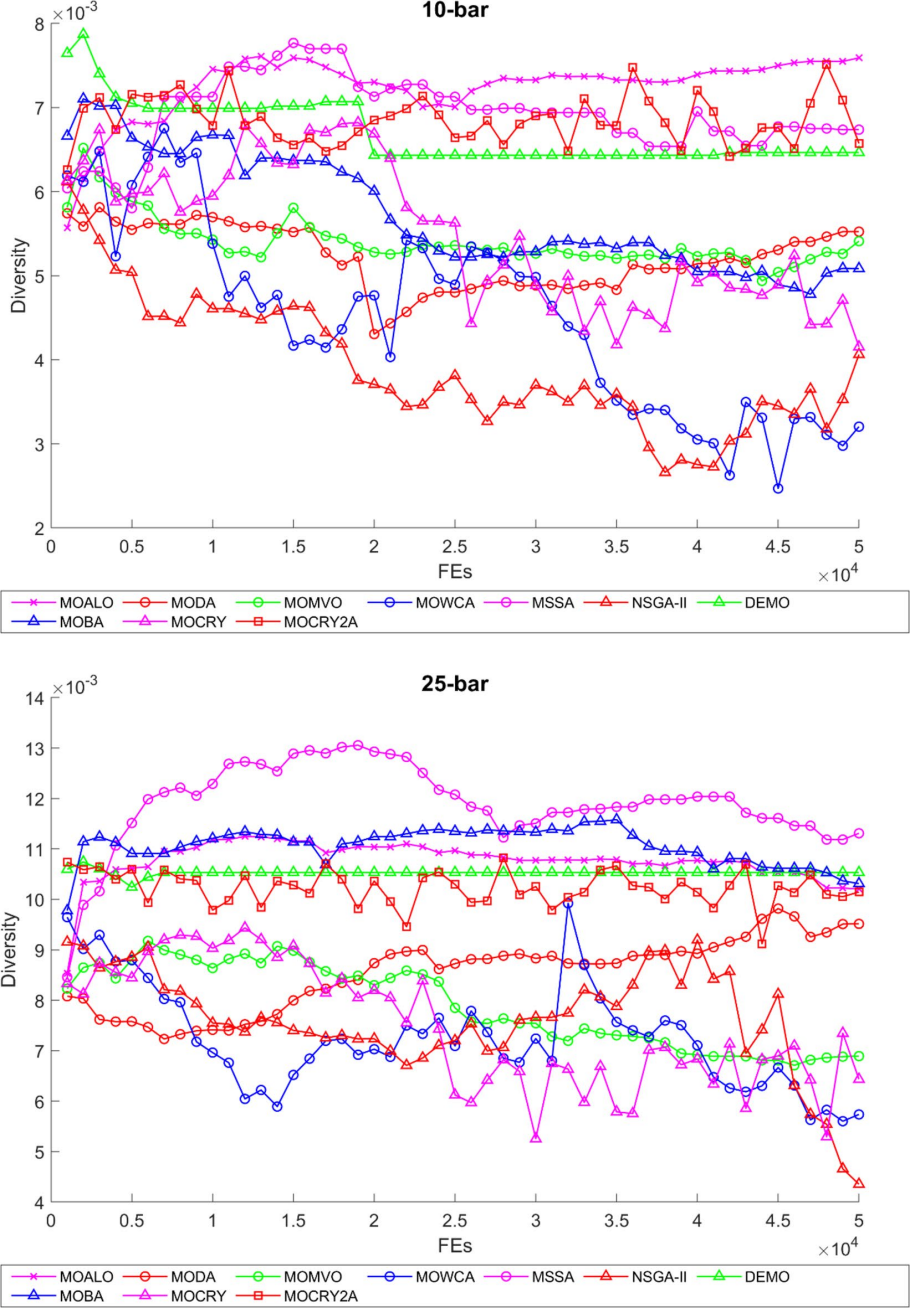

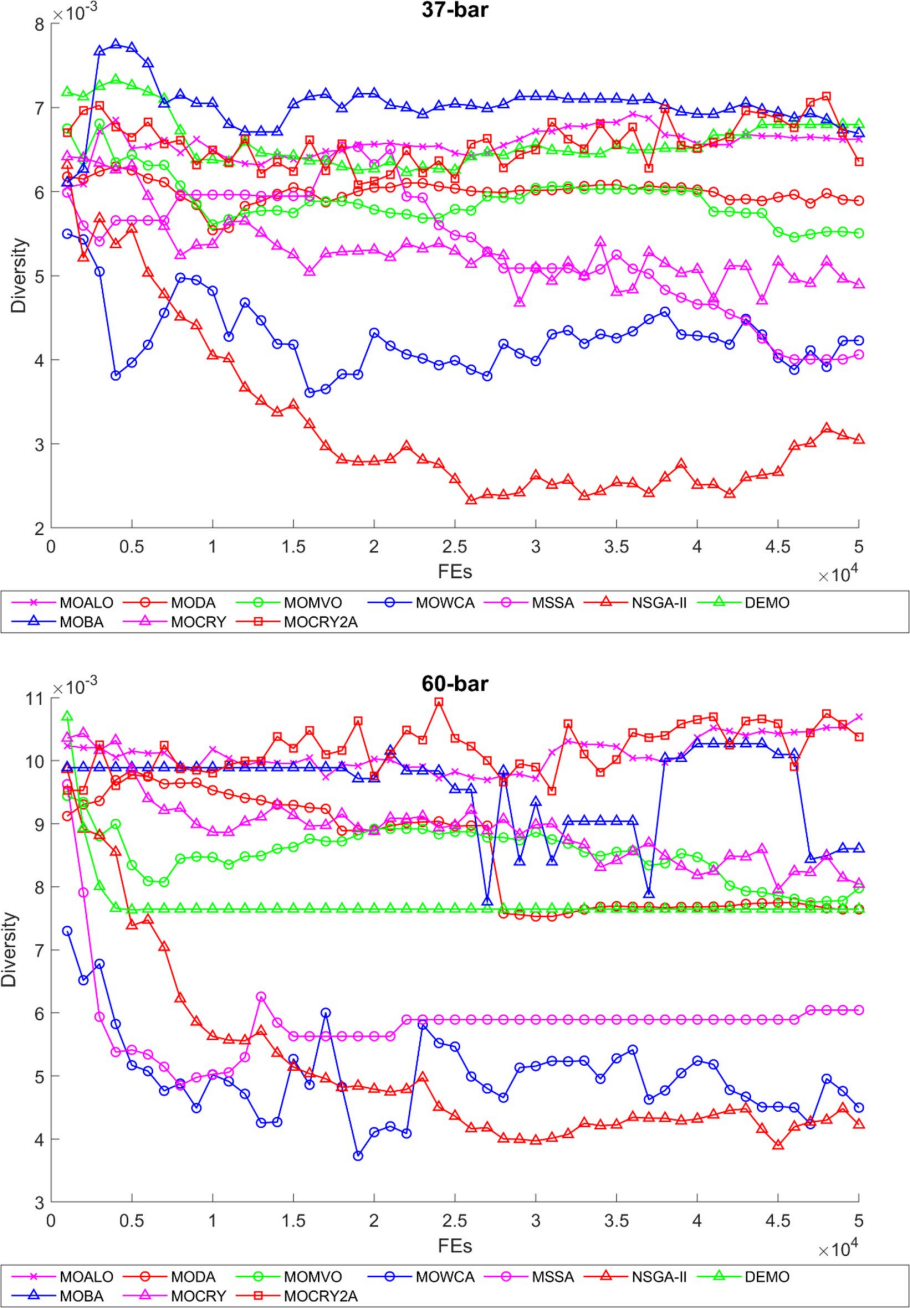

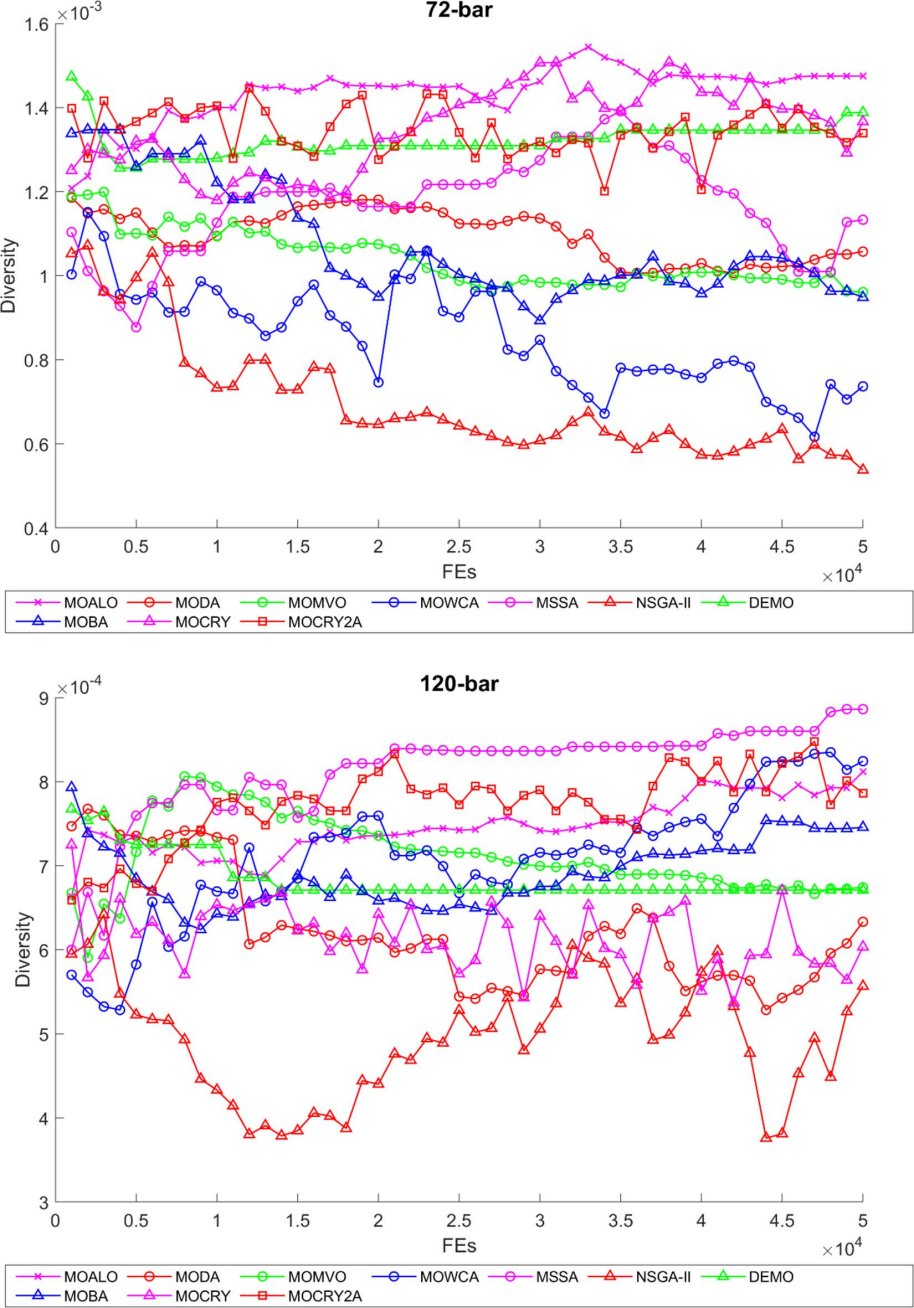

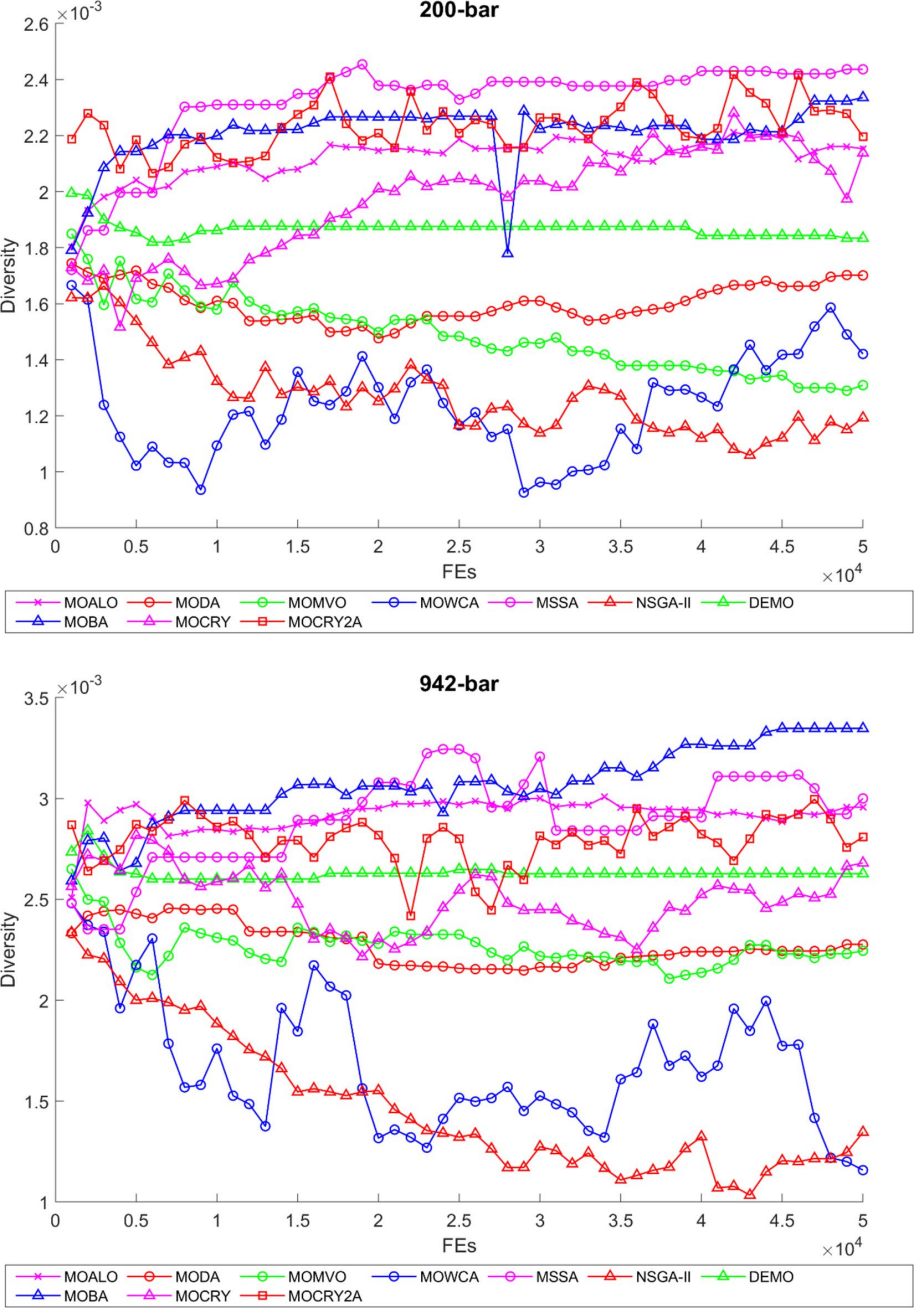
Diversity curves for 10-bar to 942-bar trusses.

**Fig. 23:**
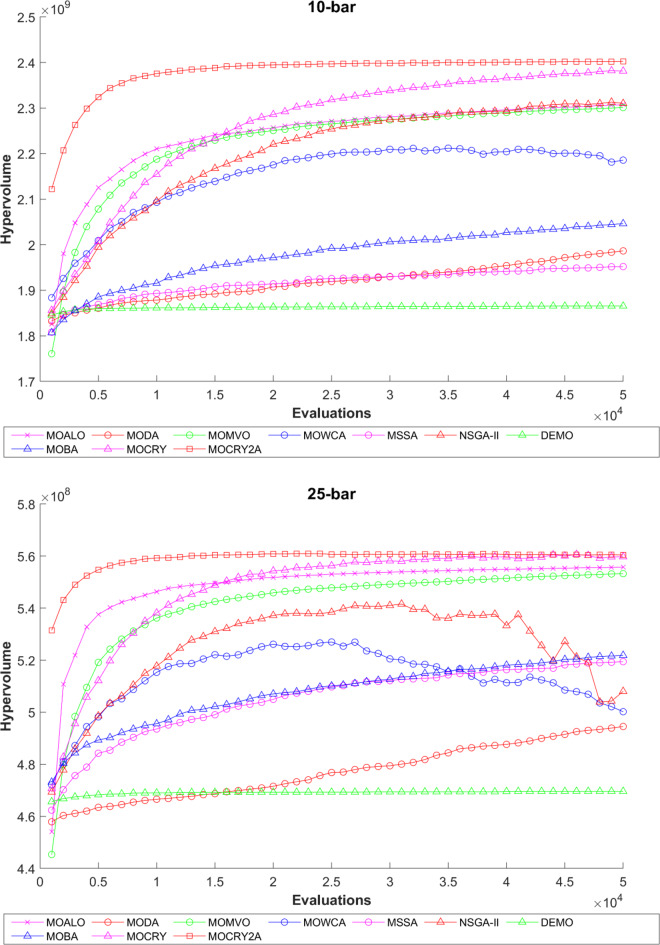

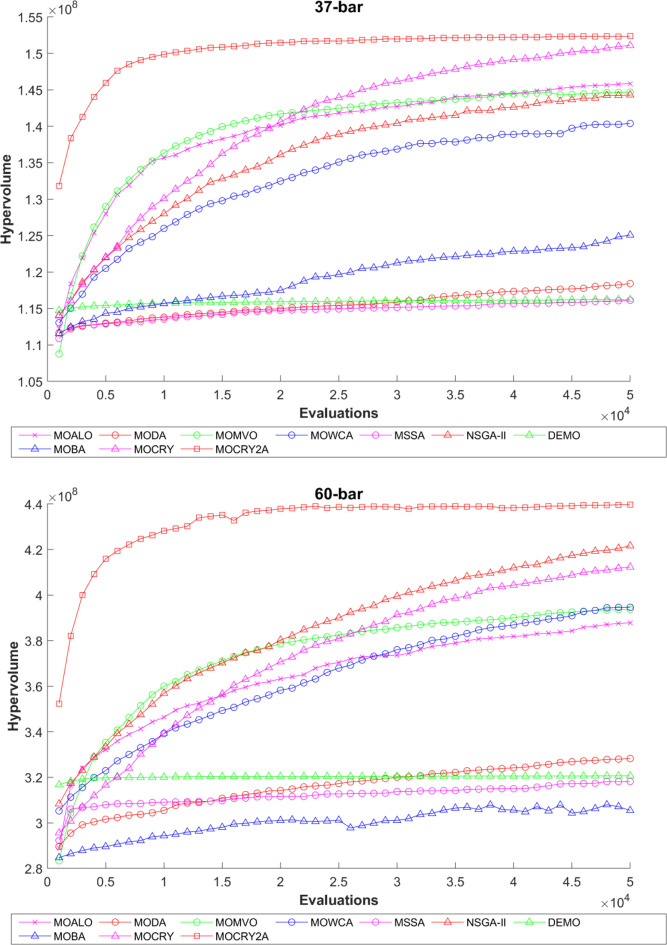

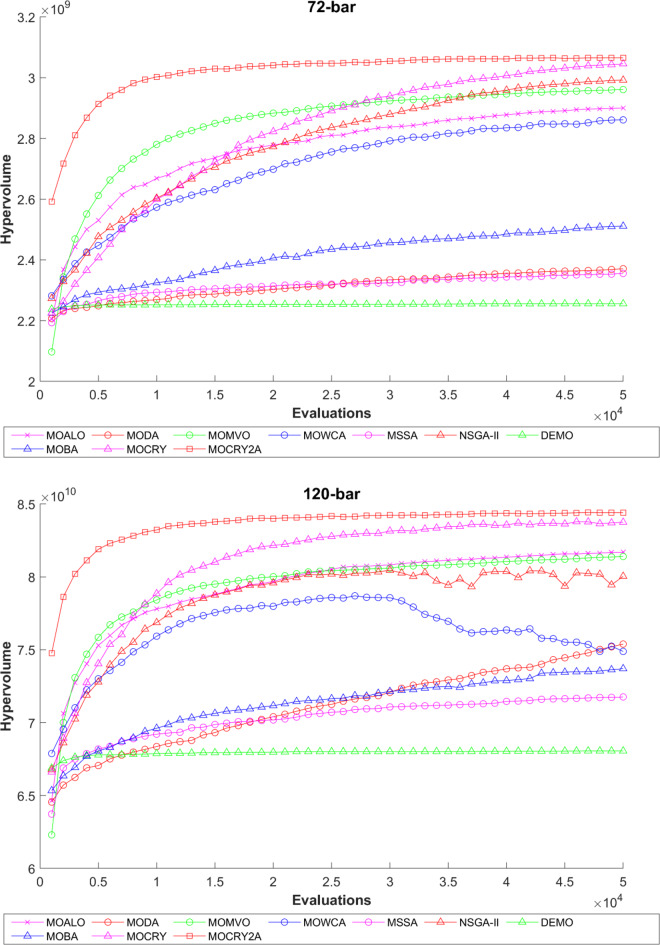

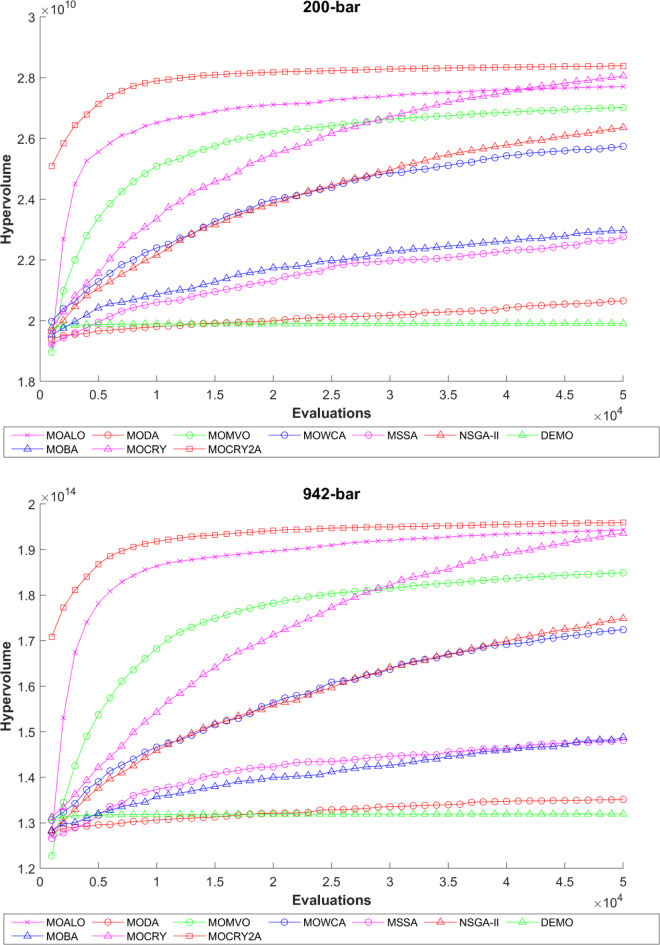
Hypervolume convergence plots.

**Fig. 24:**
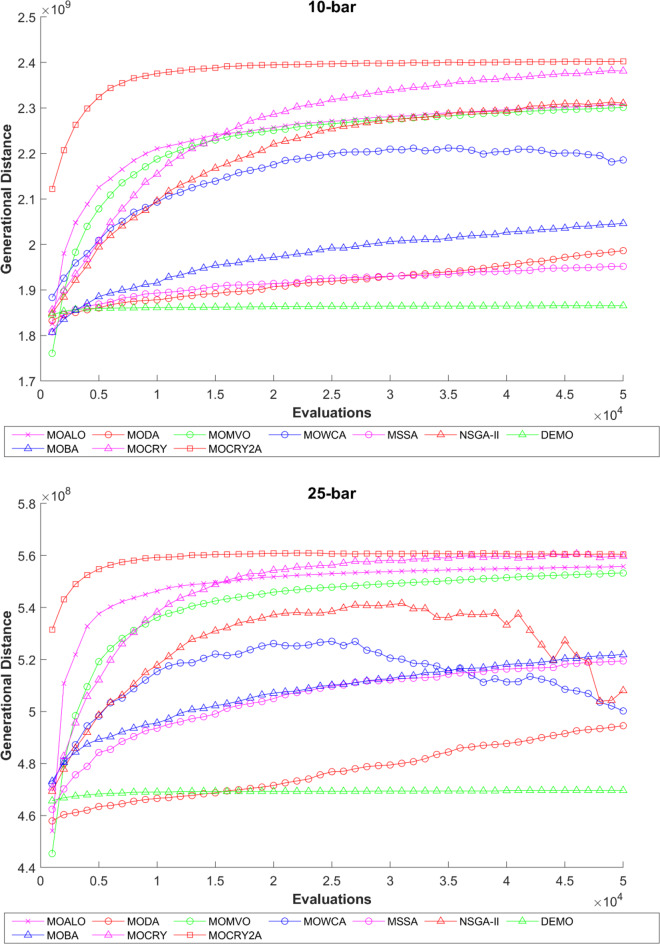

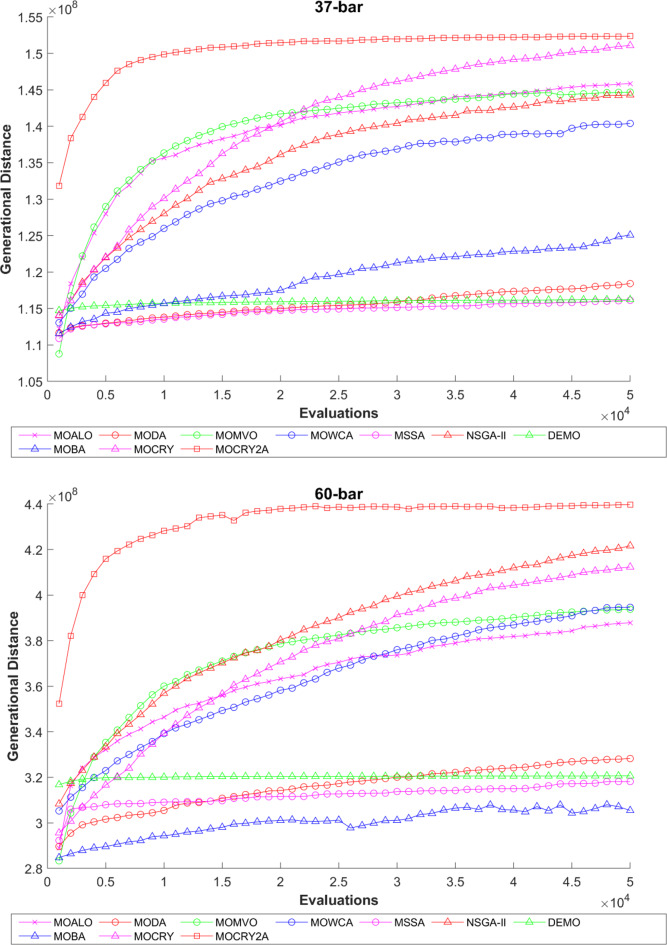

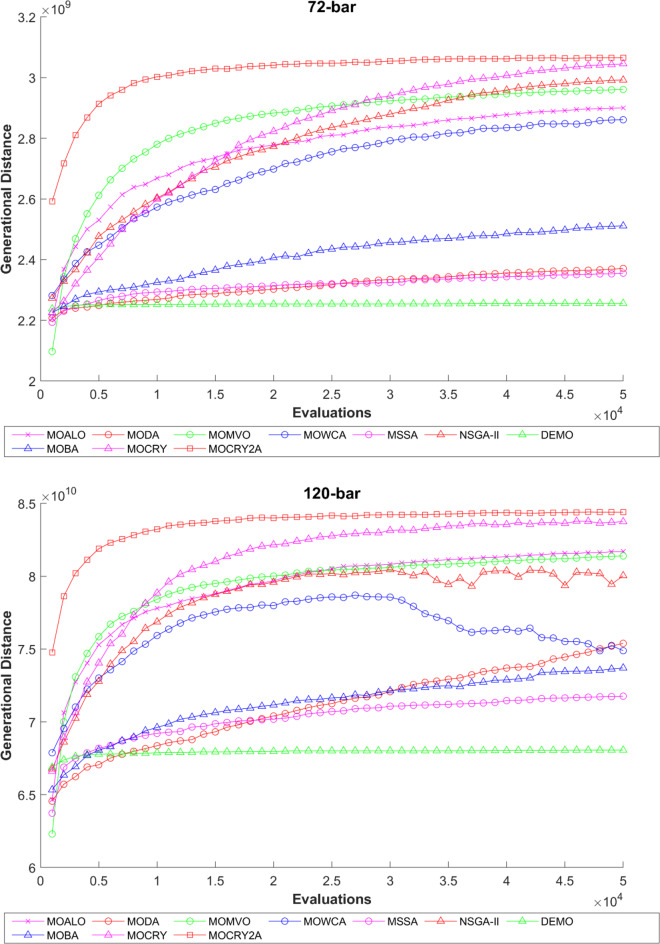

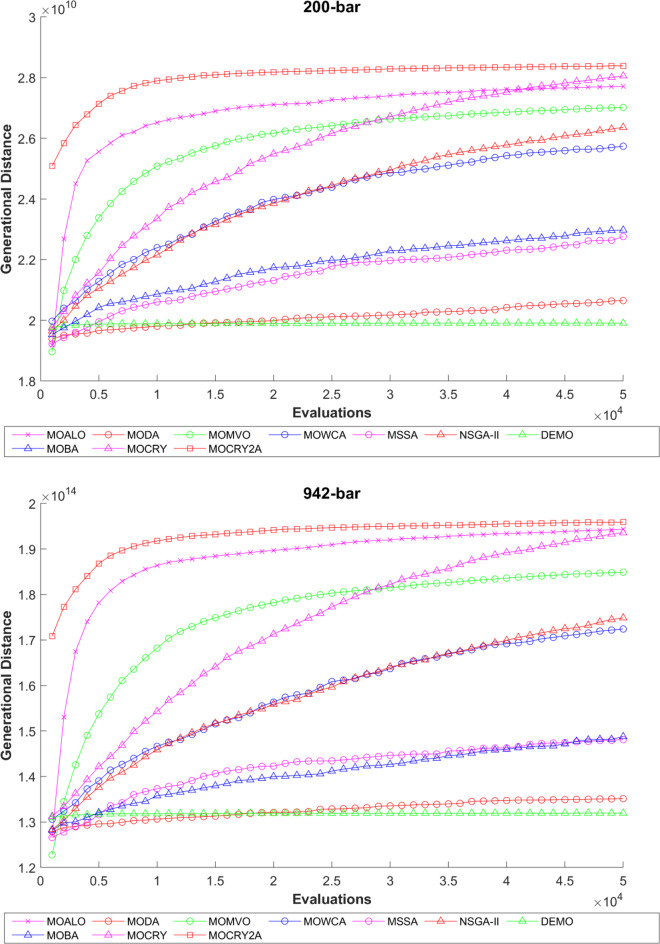
GD convergence plots.

- Table 2 records the Hypervolume (HV) results that indicate the diversity and convergence behaviour of each algorithm. Moreover, the higher the values of HV, the better the performance of the algorithm. Accordingly, MOCRY2arc realized the superior values in terms of maximum, minimum, average, standard deviation and Friedman rank test (F-rank) compared to rest of the algorithms including MOCRY. In the test, MOCRY2arc achieved an average F-rank of 1.238, placing it first overall, followed by MOCRY and MOCRY in second and third place. Moreover, for 120-, 200-, and 942-bar MOCRY2arc, it pursued the highest maximum values for fitness functions. For the 120-bar truss, MOCRY2arc achieved better results than MOALO, MODA, MOCRY, MOWCA, MSSA, NSGA-II, DEMO, MOBA, and MOCRY. Similarly, MOCRY2arc achieves an average of 3% and 5% higher maximum values for the fitness functions for the 200-bar and 942-bar trusses, respectively, when compared to all other algorithms. Moreover, MOCRY2arc retains the least standard deviation in all truss cases, demonstrating superior performance compared to other optimizers. This being said, MOCRY2arc has potential for superior Pareto fronts compared to others.
- Figures 6–13 show the Pareto front patterns obtained by MOALO, MODA, MOCRY, MOWCA, MSSA, NSGA-II, DEMO, MOBA, MOCRY, and MOCRY2arc for each of the truss problems. These graphs draw attention to the relationship between the objective functions of the problems, viz., mass and nodal displacement in the present study. As a result, the MOCRY2arc creates Pareto fronts that are evenly spread out and uninterrupted for each truss design problem. This shows that it is better than other optimizers. However, the rest of the optimizers realized slightly erratic and randomly scattered patterns for each truss structure, especially in the case of the 120-bar, 200-bar, and 942-bar. Subsequently, Figs. 14–21 show the boxplot analysis for each truss structure. This plot illustrates the trends in hypervolume distribution, indicating the superior performance of optimizers that maintain compact boxplots. In these evaluations, MOCRY2arc achieved a compact boxplot for each truss structure, outperforming the other optimizers.

- To examine the difference among NDSs and Pareto front results, the Generational Distance (GD) metric values are acquired. Table 3 records the findings for the 10-bar to 942-bar configurations of truss designs. The lower the GD value, the more superior the non-dominant front. For 10-bar truss, the GD metric standard deviation of MOCRY2arc is 3.379615; for 25-bar truss, it was 1.333073; for 37-bar truss, it was 0.655056; for 60-bar truss, it was 1.472132; for 72-bar truss, it was 2.878633; for 120-bar truss, it was 18.07715; for 200-bar truss, it was 6.960366 and for 942-bar truss, it was 1124.786. The results of the GD measure show that MOCRY2arc performs better than alternative methods. The MOBA and NSGA-II perform poorly on the GD criteria. Additionally, MOCRY2arc sustains second rank overall for Friedman’s rank at a 95% significant level. The outcomes show that the algorithms are able to preserve appropriate variety among Pareto optimum fronts.
- The IGD parameter measures both the diversity and convergence of Pareto fronts. A lower IGD value displays the superior NDS. Table 4 displays the results of the IGD measures. MOCRY2arc yields the best IGD values, with MOCRY and MOCRY following closely behind. Due to their lack of diversity, MOWCA and DEMO provide the lowest IGD values for the challenges under consideration.
- Table 5 shows the results for the STE metrics analysis, which gives the concept of spacing and the extent of Pareto fronts at the same venue. Accordingly, the lower the values of the STE metric, the better the performance of the optimizer. According to the recorded results in Table 5, MOCRY2arc achieves the lowest maximum values for the fitness functions of 10-bar, 25-bar, 37-bar, 60-bar, and 72-bar, which are 0.006796, 0.00601, 0.008455, 0.007639, and 0.009052, respectively. Subsequently, MOCRY2arc achieved 0.006344, 0.008082, and 0.006244 for the fitness functions of 120-bar, 200-bar, and 942-bar, respectively. Moreover, MOCRY2arc achieved the 1.767 and 1 average and overall F-rank, respectively, for the STE tests, which shows a clear dominance and superior performance of the algorithm. Furthermore, MOCRY, MSSA, and MOCRY achieved the second, third, and fourth overall F-rank, demonstrating competitive results with MOCRY2arc.
- During the algorithm’s execution, the diversity curves, as depicted in Fig. 22, can determine the average distance between the solutions. The results are plotted for the 50,000 functional evaluations. Moreover, MOCRY2arc shows higher diversity in the solution space compared to other optimizers. This being said, the augmentation of two archives results in effective performance for the MOCRY algorithm.
- Figs. 23 and 24 record the HV convergence and GD convergence plots of the compared algorithms for all truss configurations. Accordingly, these plots provide a potential optimizer that realizes converged results with a global optimum solution. Furthermore, the HV and GD convergence plots of the MOCRY2arc are stable and well-distributed, in contrast to the erratic nature commonly observed in DEMO, MSSA, and MOBA.

- The aggregate Friedman rank that the examined algorithms produced for each truss issue is shown in Table 6. The MOCRY2arc’s overall Friedman rank value improves when compared to NSGA-II, MOALO, MOCRY, MSSA, DEMO, MOBA, MODA, MOWCA, and MOCRY, suggesting a significant rate of convergence. The greatest and lowest average Friedman values are 2.6083 and 1.4063 for MOCRY2arc. The result tables clearly show that MOCRY2arc tops the Friedman rank overall, followed by MOCRY, MOCRY, and NSGA-II. Friedman’s rank indicates MOCRY2arc supremacy and superior performance at a 95% confidence level.

## Conclusion

The current study used MOCRY2arc, an effective version of the MOCRY optimizer enhanced with the 2-archives technique, for multiple objective optimization problems. The method used a leader selection strategy to choose solutions from the archive and a set of basic MOCRY optimizers to retain the NDS acquired. MOCRY2arc was tested on eight difficult structural optimization issues to assess its convergence, local optima avoidance, exploratory, and exploitative properties. Four commonly used performance criteria are used to compare the MOCRY2arc results with those of nine other methods. On the basis of the NDS set and their patterns close to Pareto fronts, both quantitative and qualitative studies have been carried out. One significant element that improves computational time and convergence for the MHs is the archiving approach. The average Friedman rank test grades the recommended MOCRY2arc approach highest for all technical issues. When dealing with a range of usually conflicting objectives in truss optimization, the results provide a fresh viewpoint on the benefits and drawbacks of evolutionary multi-objective optimization techniques. The proposed framework aids in the development of original solutions for real-world design optimization problems in addition to making it easier to examine them.

## Future directions and managerial implications

Further research into additional real-world critical and imperative challenges is required to evaluate MOCRY2arc’s potential, even if it shows efficiency in the design problems studied. The interested researcher can expand this study to include functional technical issues with several conflicting aims that are multimodal and multidimensional. The research study details numerous strategies for enhancing MOCRY’s performance.

The proposed algorithm can be further applied to globally optimize multiple objective challenges across the multidisciplinary domains. Accordingly, not limited but including to, mechanical engineering design optimization, fuzzy logic circuits optimization, work and batch scheduling optimization, automobile system optimization and parametric optimization of solar photovoltaic panels^41^.

## Data Availability

The datasets used and analyzed during the current study are available from the corresponding author upon reasonable request.
